# Proteome Analysis Identified the PPARγ Ligand 15d-PGJ2 as a Novel Drug Inhibiting Melanoma Progression and Interfering with Tumor-Stroma Interaction

**DOI:** 10.1371/journal.pone.0046103

**Published:** 2012-09-25

**Authors:** Verena Paulitschke, Silke Gruber, Elisabeth Hofstätter, Verena Haudek-Prinz, Philipp Klepeisz, Nikolaus Schicher, Constanze Jonak, Peter Petzelbauer, Hubert Pehamberger, Christopher Gerner, Rainer Kunstfeld

**Affiliations:** 1 Department of Dermatology, Medical University of Vienna, Vienna, Austria; 2 Department of Medicine I, Medical University of Vienna, Vienna, Austria; The Moffitt Cancer Center & Research Institute, United States of America

## Abstract

Peroxisome proliferator-activated receptors (PPARs) have been originally thought to be restricted to lipid metabolism or glucose homeostasis. Recently, evidence is growing that PPARγ ligands have inhibitory effects on tumor growth. To shed light on the potential therapeutic effects on melanoma we tested a panel of PPAR agonists on their ability to block tumor proliferation *in vitro*. Whereas ciglitazone, troglitazone and WY14643 showed moderate effects on proliferation, 15d-PGJ2 displayed profound anti-tumor activity on four different melanoma cell lines tested. Additionally, 15d-PGJ2 inhibited proliferation of tumor-associated fibroblasts and tube formation of endothelial cells. 15d-PGJ2 induced the tumor suppressor gene p21, a G_2_/M arrest and inhibited tumor cell migration. Shot gun proteome analysis in addition to 2D-gel electrophoresis and immunoprecipitation of A375 melanoma cells suggested that 15d-PGJ2 might exert its effects via modification and/or downregulation of Hsp-90 (heat shock protein 90) and several chaperones. Applying the recently established CPL/MUW database with a panel of defined classification signatures, we demonstrated a regulation of proteins involved in metastasis, transport or protein synthesis including paxillin, angio-associated migratory cell protein or matrix metalloproteinase-2 as confirmed by zymography. Our data revealed for the first time a profound effect of the single compound 15d-PGJ2 on melanoma cells in addition to the tumor-associated microenvironment suggesting synergistic therapeutic efficiency.

## Introduction

Defining novel treatment options of melanoma is still a challenge and the identification of new agents is vital due to the increasing incidence and poor prognosis [1], [2]. For any novel drug, many obstacles have to be overcome from target identification to clinical testing of therapeutics. Therefore, drugs already approved for the treatment of other diseases but potentially applicable in melanoma are of high interest [1].

There is increasing evidence that the peroxisome proliferator-activated receptor-γ (PPARγ)-binding ligands, may be effective for the treatment of melanoma [1] and other tumors [3].

PPARs are ligand-activated transcription factors of the nuclear hormone receptor superfamily comprising three subtypes: PPARα, PPARγ, and PPARδ/β and are characterized by distinct functions, ligand specificities and tissue distribution [4]. The role of these receptors has been considered originally to be restricted to lipid and lipoprotein metabolism, glucose homeostasis and cellular differentiation [5].

PPARγ was demonstrated to regulate diverse cellular and neoplastic processes such as proliferation [6], differentiation [7] and apoptosis [8]. The anti-tumor effect of PPARγ activation is exerted by the induction of cell cycle arrest rather than by induction of apoptosis [9], [10]. In addition, the inhibition of endothelial cell migration by PPARγ ligands has been described, bolstering the anti-angiogenic activity of PPAR ligands [11], [12].

The PPARγ specific agonists 15-deoxy-Δ12,14 prostaglandin J2 (15d-PGJ2), troglitazone, and rosiglitazone inhibited cell proliferation in four melanoma cell lines dose-dependently, whereas a specific agonist of peroxisome proliferator-activated receptor alpha (WY-14643) did not exert this effect [9]. Ciglitazone, a selective PPARγ ligand, was shown to inhibit the proliferation of the A375 as well as of the WM35 melanoma cell line [13].

Several PPAR ligands are interesting candidates for melanoma therapy. Thiazolidinediones (TZD), ciglitazone and troglitazone are high affinity synthetic ligands. In contrast, 15d-PGJ2 is a low-affinity endogenous ligand for PPARγ and known to be a potent inducer of heme oxygenase 1 (HO-1). The three high affinity ligands directly regulate cyclin D1 and p21 and the multi-functional protein ß-catenin [14], [15]. The latter observation implies that PPARγ ligands may be able to interfere with the metastatic process [16].

Here we present a comprehensive study assessing the anti-tumorigenic effects of a panel of PPARα and PPARγ agonists on a variety of melanoma cell lines. The PPARγ agonists ciglitazone, troglitazone and 15d-PGJ2 and the PPARα ligand WY-14643 were tested on four melanoma cell lines (A375, M24met, 1205Lu and MelJuso) to generalize our findings. In addition to direct effects on cancer cells, PPARγ agonists were tested on the influence on cells of the tumor microenvironment such as endothelial cells and melanoma associated fibroblasts.

To further investigate molecular mechanisms of drug action we made use of the proteome profiling methods shot gun analysis and 2D-gel electrophoresis. Applying the recently established CPL/MUW proteomics database [17], [18] we were able to detect protein alterations independently supporting the present functional data.

Our study indicates that 15d-PGJ2 is a potent anti-tumorigenic compound by interfering with melanoma cell proliferation, metastasis and additionally affecting the melanoma associated stroma.

## Results

### 15d-PGJ2 inhibits cell proliferation more efficiently than other PPAR ligands via cell cycle arrest and p53 regulation

We investigated the anti-proliferative effects of PPARγ ligands ciglitazone, troglitazone and 15d-PGJ2 and the PPARα ligand WY-14643 on four melanoma cell lines (A375, M24met, 1205Lu and MelJuso). As determined by MTS proliferation assays, the IC_50_ of 15d-PGJ2 was in a range between 22–38 µM after 48 h of treatment (Table S1). In contrast the IC_50_ of the PPARγ agonists ciglitazone and troglitazone could not be reached with the highest dose of 100 µM tested on A375, M24met and MelJuso melanoma cell lines. The selective PPARα agonist WY-14643 showed no growth inhibitory effect (Table S1). Thus, among the tested PPARγ agonists 15d-PGJ2 was found most efficient.

Next we investigated the anti-proliferative effects on human umbilical vein endothelial cells (HUVECs) and skin-derived fibroblasts of healthy donors. The IC_50_ of isolated HUVECs was 85, of LECs 70.84, suggesting a restriction of 15d-PGJ2 efficiency to malignant cells (Table S1).

In contrast to normal fibroblasts such as NHDF with an IC50 of 127.70, the melanoma associated fibroblasts of four different patients revealed to be more sensitive upon15d-PGJ2 treatment (IC_50_ range: 44–68 µM).

**Figure 1:**
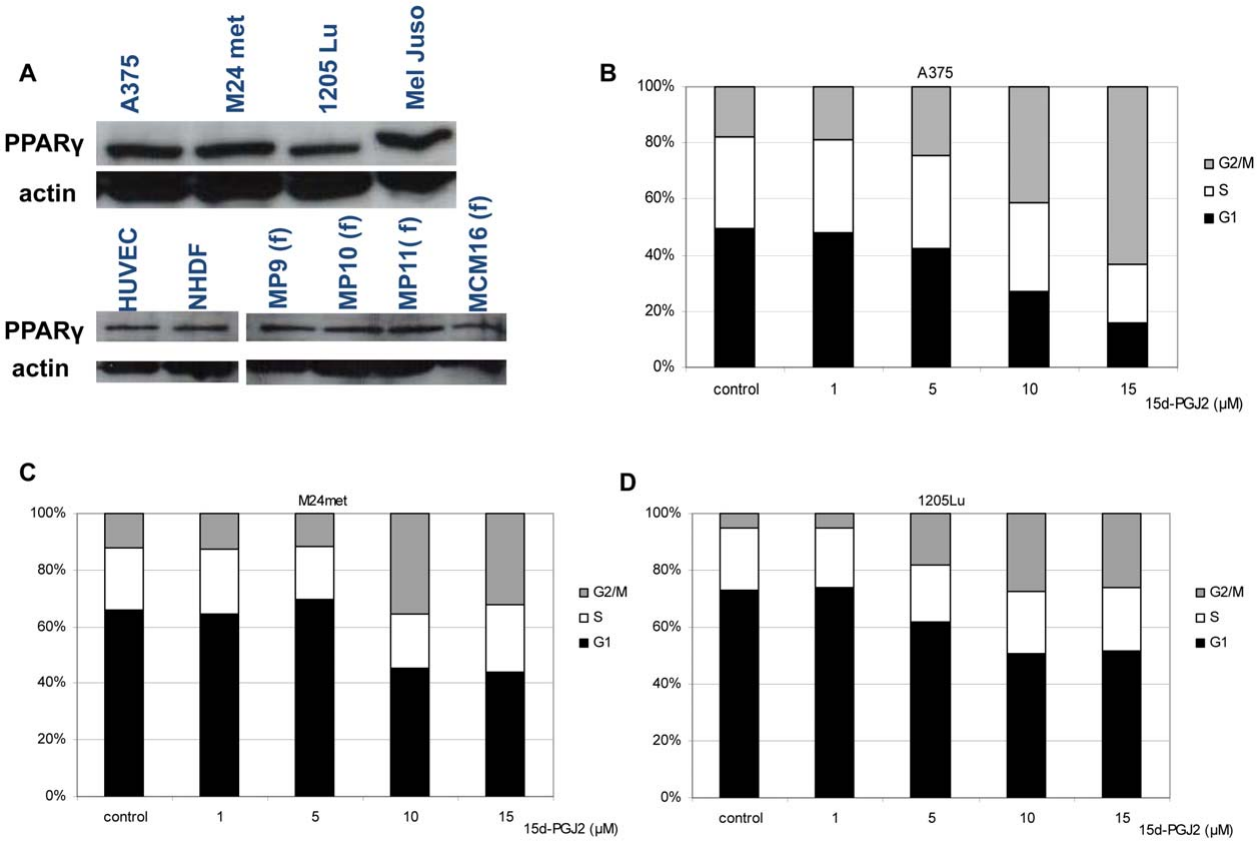
PPARγ receptor expression and G2/M arrest induction by 15d-PGJ2. A, receptor expression on A375, M24met, 1205Lu, MelJuso melanoma cells and HUVECs as well as fibroblasts NHDF and melanoma associated fibroblasts. (f): fibroblasts B, C ,D: Cell cycle analysis by flow cytometry using propidium iodide- stained on A375, M24met and 1205Lu. Cells were treated for 24 h with different concentrations of 15d-PGJ2. Three independent experiments were pooled and analyzed as a combined data set.

The PPARγ expression in the melanoma cell lines (A375, M24met, 1205Lu, MelJuso), in HUVECs, normal fibroblasts (NHDFs) and primary melanoma associated fibroblasts (MP9, MP10, MP11, MCM16 fibroblasts) was confirmed via Western blotting (Fig. 1A).

We selected 15d-PGJ2, the most potent PPARγ agonist for further investigations.

We analyzed cell cycle alterations mediated by 15d-PGJ2 in A375, M24met and 1205Lu melanoma cell lines. In all melanoma cell lines 15d-PGJ2 induced a G_2_/M arrest. Treatment of cells with 15 µM 15d-PGJ2 triggered cell cycle arrest in the G_2_/M phase from 18 to 63 percent, from 12 to 32 percent in and from 5 to 26 percent in A375 (Fig. 1B), in M24met (Fig. 1C) and in 1205Lu cells (Fig. 1D), respectively.

**Figure 2:**
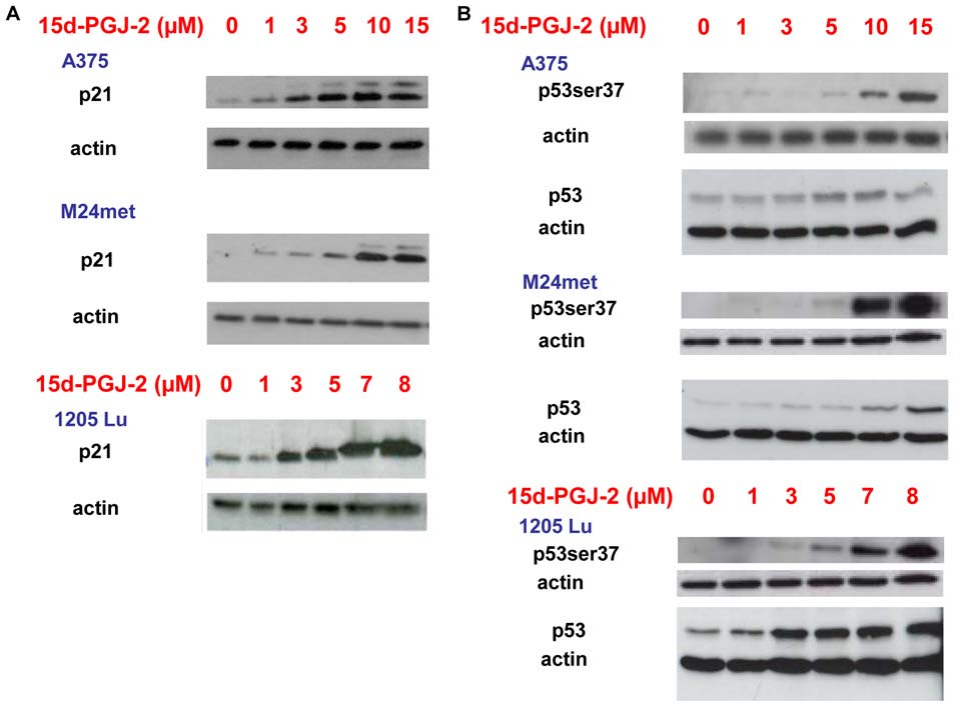
15d-PGJ2 induces p21 expression and p53, p53ser37. A, 15d-PGJ2 leads to an induction of p21. Immunoblotting of p21 after 48 hours of 15d-PGJ2 treatment with indicated concentrations. B, Immunoblotting of p53 and p53ser37 after 48 hours of 15d-PGJ2 treatment with indicated concentrations.

Since p21 is known to induce S-phase or G_2_/M arrest [19], [20], [21], we tested our cells for p21 induction after 15d-PGJ2 treatment. Indeed, 15d-PGJ2 treatment dose- dependently induced upregulation of p21 in A375, M24met and 1205Lu at low micromolar concentrations (Fig. 2A). Additionally, 15d-PGJ2 induced p53 expression and/or phosphorylation in A375, M24met and 1205Lu melanoma cell lines (Fig. 2B).

### 15d-PGJ2 exerts inhibitory effects on tumor cell migration, angiogenesis and lymphangiogenesis

**Figure 3:**
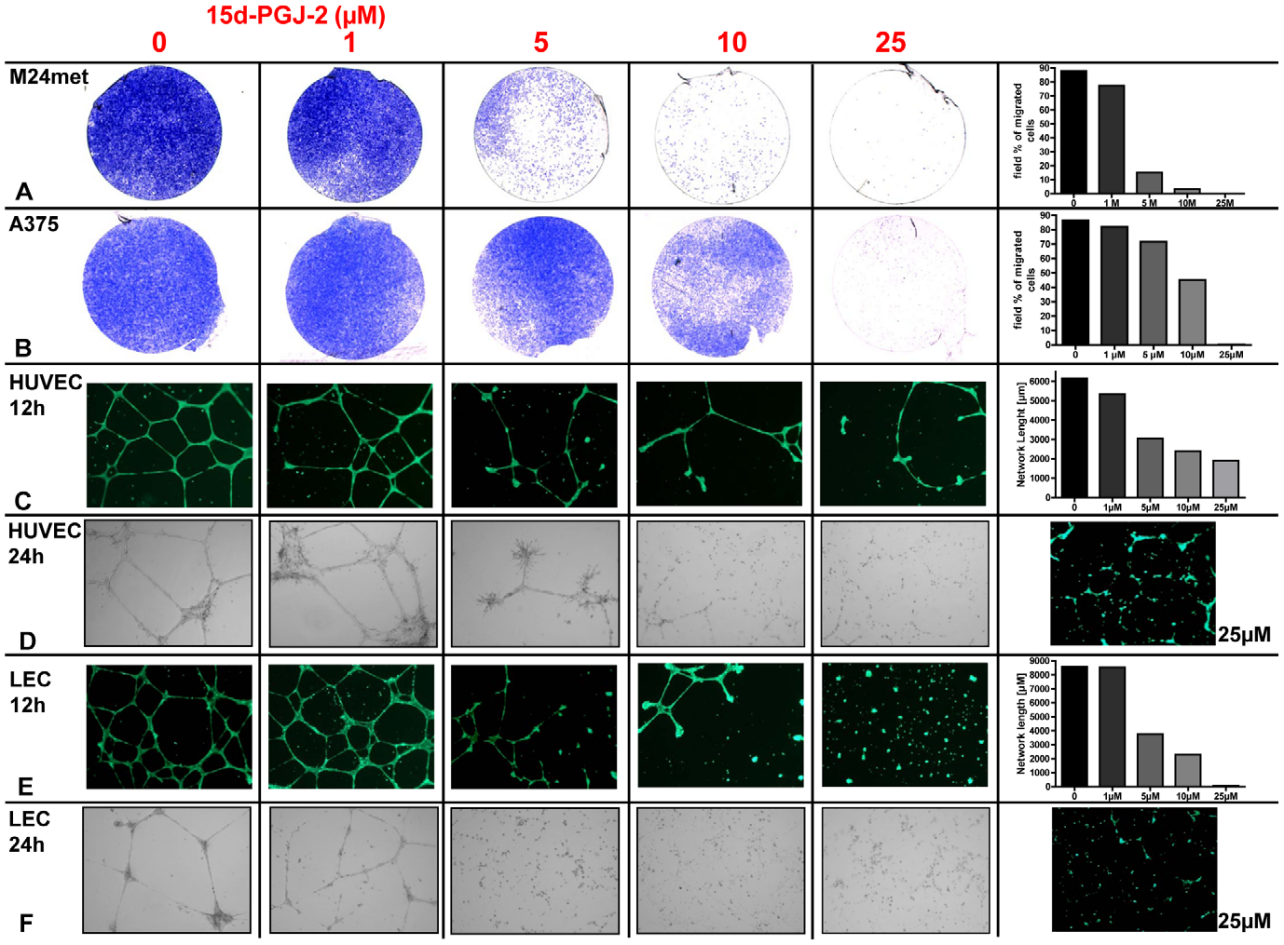
15d-PGJ2 inhibits tumor cell migration and tube formation of HUVECs and LECs. A, B, Tumor cell migration assay after 48 hours of M24met melanoma cells and A375 melanoma cell line treated with 15d-PGJ2 with indicated concentrations. Representative pictures of three independent experiments. Quantification of the depicted experiment is performed using Axiovision Software. C–F, tube formation assay of HUVECs and LECs with indicated concentrations after 24 and 48 hours. Calcein staining was performed to monitor the vitality of the cells. Tube formation was quantified using Cell Profiler Software Package. Representative pictures of three independent experiments.

Impact of 15d-PGJ2 on melanoma cell migration was investigated using a Matrigel invasion chamber assay. 15d-PGJ2 inhibited M24met melanoma cell migration in a dose-dependent manner and inhibited tumor cell migration at a concentration of 5 µM after 48 hours (Fig. 3A). At a concentration of 25 µM migration is totally abolished as demonstrated in the M24met and A375 melanoma cell lines (Fig. 3 A, B). The percentage of transmigrated cells is quantified by Axiovion software.

Inhibition of angiogenesis was demonstrated by a dose dependent disturbance of tube formation of HUVECs after 12 and 24 hours (Fig. 3C, D). Inhibition of lymphangiogenesis was indicated repeating these experiments with lymphatic endothelial cells (LECs) (Fig. 3E, F). Here pronounced effects could be observed already at a concentration of 5 µM 15d-PGJ2. Tube formation was quantified using the Cell Profiler Software Package and calcein staining was used to demonstrate the vitality of the cells.

### Shot gun analysis for characterisation of the acting profile of 15d-PGJ2

**Figure 4:**
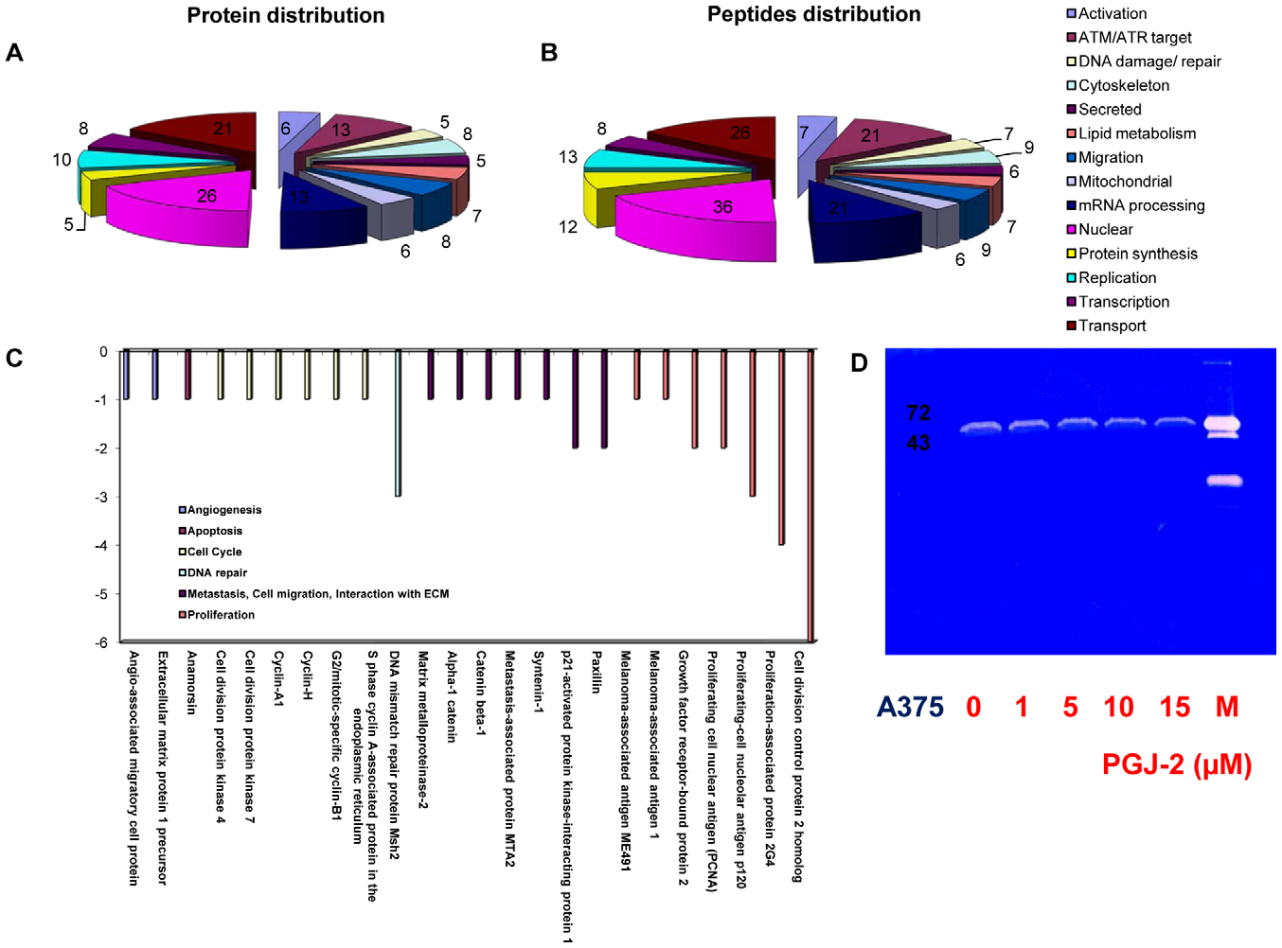
Shot gun analysis and Zymography of A375 melanoma cells treated with 15d-PGJ2. A, B classification of all induced proteins and peptides by the bioinformatic database of A375 melanoma cells treated with 5 µM 15d-PGJ2 for 48 hours. C, downregulated proteins by 5 µM 15d-PGJ2 identified by shot gun analysis. The legend depicts the classification of the identified proteins. D, Zymography assay of the supernatant of A375 melanoma cells treated with 1, 5, 10, 15 µM 15d-PGJ2 for 48 hours.

Shot gun analysis of nuclear and cytoplasmic fractions of untreated A375 cells resulted in the identification of a total of 2250 proteins. Proteins were classified according to gene ontology terms accessible via uniprot. Shot gun analysis of 15d-PGJ2-treated A375 cells revealed 136 proteins which displayed increased peptide counts compared to the control (Table S2A, B). Amongst these we identified proteins involved in the lipid metabolism (protein count/peptide count = 7/7) such as thromboxane-A synthase, adipophilin, perilipin or apolipoprotein A-I (Table S2A, B) [22], [23]. Additionally, we detected the induction of HO-1 by 15d-PGJ2 (Table S2A, B) [24]. As depicted in Fig. 4 A and B proteins/peptides involved in DNA repair mechanisms (5/7) such as MSH3, telomeric repeat-binding factor 2 or MMS2 (Table S2A, B), phosphorylation by ATM/ATR upon DNA damage (13/21), transport (21/26), mRNA processing (13/21), protein synthesis (5/12), replication (10/13) and transcription (8/8) were upregulated. In accordance with our data proteins involved in cell cycle such as the lymphokine-activated killer T-cell-originated protein kinase or with anti-proliferative effects such as nodal-modulator 1 revealed to be induced (Table S2A, B) (Uniprot). Proteins indicating a cellular stress response such as sterile 20/oxidant stress-response kinase 1 or growth arrest and DNA damage-inducible protein GADD45 beta were regulated as well (Table S2A, B) (Uniprot). The DNA repair proteins MMS2, MSH3, MSH6, MSH2, MLH1 and the upregulation of basigin at 1 µM and nodal at 15 µM was confirmed by Western blot analysis (Fig. S1).

Furthermore, several proteins related to angiogenesis such as angio-associated migratory cell protein, to cell cycle such as cyclin – A1 and H, to metastasis, to cell migration and to interaction with the extracellular matrix (ECM) such as paxillin or syntenin-1 and to proliferation such as PCNA (proliferating cell nuclear antigen) (Fig. 4C, Table S3) (Uniprot) were found to be downregulated .Evidence is growing that PPARγ ligands may be potent inhibitors of matrix metalloproteinases (MMPs) such as MMP 2, 7 and 9 [25], [26], [27], [28], [29]. The present shot gun proteomics data demonstrate downregulation of MMP 2, a key player in the metastatic process (Fig. 4C, Table S3). Employing a zymography assay we confirmed the downregulation of MMP2 by 15d-PGJ2 (Fig. 4D). To exclude unspecificity and cytotoxic side effects we evaluated if 15d-PGJ2 exhibits effects on the NF-kappa-B pathway. In the shotgun data we did not observe upregulation of constituents of the NF-kappa-B signalling pathway such as I-kappa-B-kinase 2 or NF-kappa-B inhibitor-interacting Ras-like protein 2 (Kappa B-Ras protein 2). In addition we observe no upregulation of NF-kappa-B by western blot analysis (data not shown).

### 15d-PGJ2 highly downregulates a panel of chaperones and leads to a modification of Hsp90 in 2D-gel electrophoresis

**Figure 5:**
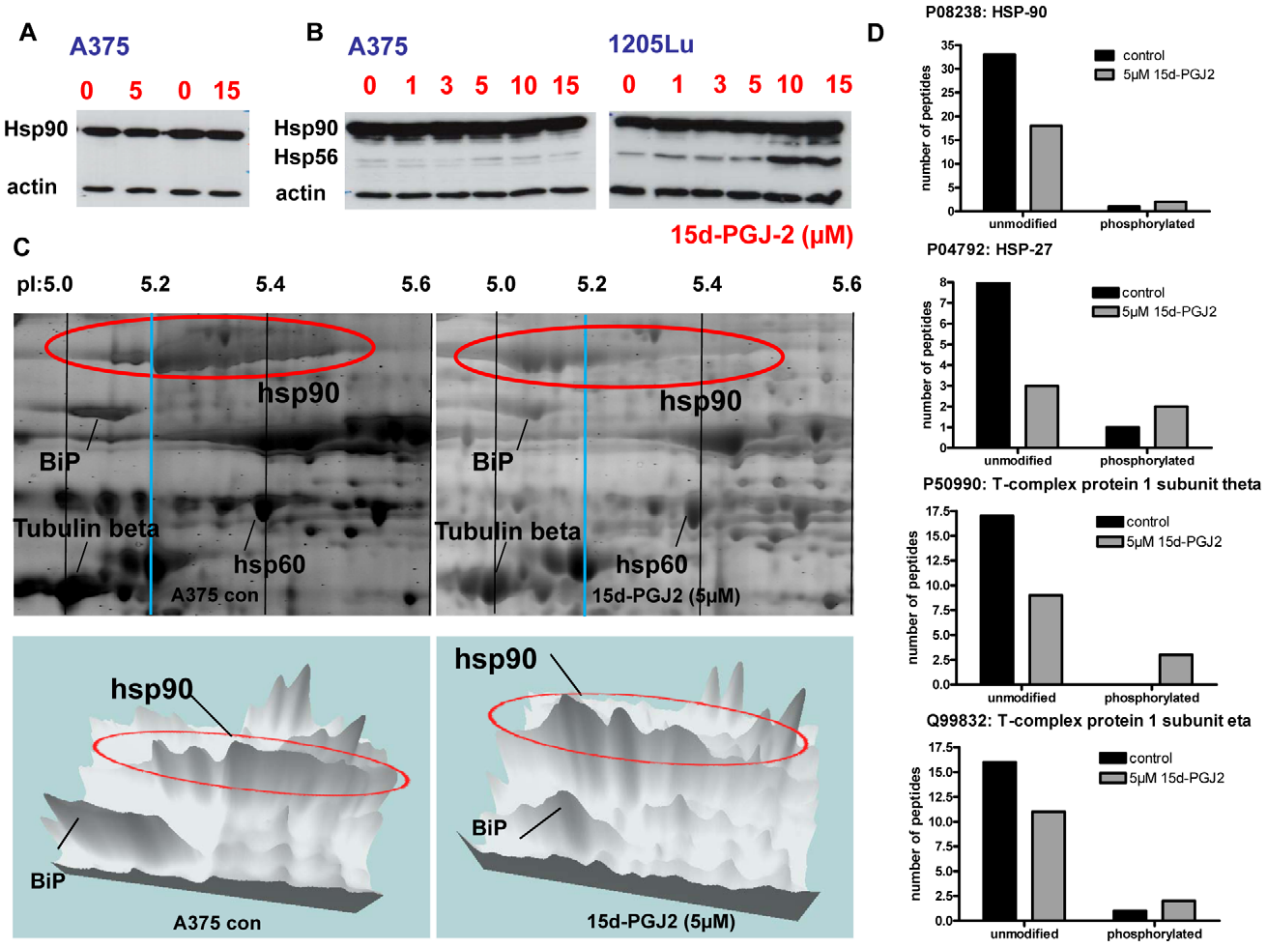
pI shift of Hsp90 in 2D-gel electrophoresis and 15d-PGJ2 induced phosphorylation of several chaperones. A, Representative immunoblots of three independent experiments of Hsp90 cytosolic (A375) and B, total cell lysate (A375 and 1205Lu). C, 2D-gel electrophoresis of 15d-PGJ2 or DMSO treated A375 melanoma cells with additional three dimensional versions. D, For immunoprecipitation, an anti-phosphoserine antibody was applied to cytoplasmic protein fractions. In case of the four listed chaperones, an apparent down-regulation observed by shotgun proteomics was actually accompanied by phosphorylation as evidenced by increased binding to anti-phosphoserine antibody. Protein abundances were estimated from the number of distinct peptides identified per protein.

A large group of 33 out of 38 detectable chaperones were downregulated (Table S4). Especially Hsp90 beta (−15) and alpha (−13) revealed to be the most prominent downregulated chaperones upon 15d-PGJ2 treatment (Table S4). However, Western blotting of the cytoplasmic fractions and total cell lysates of A375 and 1205Lu cells did not verify these results (Fig. 5A, B). Using the total cell lysate again no regulation of Hsp90 could be verified in A375 and 1205Lu melanoma cell lines, only an induction of the additional appearing band of Hsp56 (Fig. 5B).

To further investigate these surprising results we performed 2D-gel electrophoresis with cytoplasmic proteins of A375 melanoma cells. Intriguingly, Hsp90 displayed a profound pI shift from 5.2–5.4 in the control group to 5.0–5.2 upon 15d-PGJ2 treatment (Fig. 5C). This indicates posttranslational modifications of Hsp90 which may cause interference with the identification of peptides by shot gun analysis. This shift is visualized also in a 3 dimensional version of the 2D-gel (Fig. 5C).

Protein modification may result in apparent down-regulation of the number of identified peptides, because modified peptides may fail to be identified by mass spectrometry. Therefore, we further investigated protein phosphorylation by immunoprecipitation using an anti-phosphoserine antibody. Actually, all chaperones identified in the immunoprecipitates were found at increased levels in the 15d-PGJ2 treated samples (Hsp90, Hsp27,T-complex protein 1 subunit theta and eta), indicating 15d-PGJ2-induced phosphorylation (Fig. 5D). These chaperones were found to be apparently down-regulated by 15d-PGJ2 (Table S4), suggesting that partially the down-regulation observed by shotgun proteomics is accompanied by phosphorylation.

## Discussion

This study was designed to investigate consequences of PPARγ activation for melanoma and melanoma-associated stroma cells. While recent reports indicate antiproliferative effects of these drugs in several cancer cells including melanoma, this is the first investigation of PPARγ ligand effects including both melanoma cells as well as melanoma-associated stroma cells such as fibroblasts and endothelial cells.

We demonstrated that 15d-PGJ2 is much more effective compared to other PPARγ ligands in inhibiting growth of melanoma cell lines, while the PPARα ligand WY-14643 had hardly any effect. These results are in line with recent data of other laboratories [3]. Therefore we restricted subsequent analyses to 15d-PGJ2.

Prakash et al demonstrated that 15d-PGJ2 induces cell death in B16F10 melanoma and addition of serum leads to a tolerance to 15d-PGJ2 by rapidly binding to albumin [30].

Our results support previous reports of PPARγ agonists describing both a direct anti-tumor and a broad spectrum of anti-stromal, anti-angiogenetic and immuno-modulating activities [29].

Analysis of 15d-PGJ2 effects on melanoma-associated fibroblasts revealed substantial anti-proliferative effects. This finding points to a distinct effect of 15d-PGJ2 on cells in the tumor microenvironment, as normal fibroblasts did not show such a drug response.

Besides fibroblasts, endothelial cells are important players in the tumor microenvironment. Here we demonstrate that 15d-PGJ2 effectively abolished tube formation of HUVECs, which is in line with the observations that HUVEC differentiation into tube-like structures in three-dimensional collagen gels could be suppressed by specific PPARγ ligands [31]. Another anti-angiogenic mechanism is the induction of apoptotic cell death in endothelial cells after incubation with 15d-PGJ2 [32], [33]. In contrast to these data, we observed a rather high IC50 of HUVECs for 15d-PGJ2, suggesting that 15d-PGJ2 specifically interferes with the tube formation process. Since tube formation was inhibited already at a concentration of 5 µM and the cells were demonstrate to be still vital with the highest concentration tested, while the IC50 was found to be 85 µM, the destruction of the HUVEC tube formation is apparently not a result of growth inhibitory effects of 15d-PGJ2. This interpretation is supported by the finding that15d-PGJ2 transiently inhibits the expression of VEGFR-1 and VEGFR-2 [34].

The effect of 15d-PGJ2 on lymphatic endothelial cells has not been analyzed so far. In this study we provide evidence that 15d-PGJ2 also exerts anti-lymphangiogenic activity. The ability to promote lymphangiogenesis enhances the metastatic spread of melanoma and recent studies revealed that tumor associated lymphangiogenesis is significantly correlated with poor disease-free and overall survival of patients with cutaneous melanoma [35], [36]. The mechanism how, 15d-PGJ2 leads to an inhibition of lymphangiogensis has to be elucidated in further studies, since this activity adds to its potential as a therapeutic tool.

Tumor initiation and progression is associated with the transition of normal stroma into an “activated” stroma phenotype. These tumor-associated, genetically still intact cells are able to establish a supportive environment for tumor cell survival and growth and to facilitate invasion and metastasis. Targeting this interference between tumor and stroma may consistently lead to a reduction of tumor growth and metastasis. Such a therapeutic approach has been presented as biomodulatory treatment both by our group and others [37], [38] and may complement standard chemotherapeutic approaches.

In search for alternative strategies for the treatment of metastatic neoplasm, targeting the tumor stroma seems to be a promising strategy since this approach is not directly cytotoxic but interferes with the cooperativity of tumor and stroma cells [37], [38], [39]. Considering that the stroma provides proteins supporting tumor survival, a blockage of this process might chemosensitize the tumor cells. Here we showed for the first time that the receptor is expressed on a panel of melanoma associated fibroblasts while to a lower extent on normal fibroblasts such as TF. PPARγ expression in metastatic melanoma was shown to be a possible predictive marker for response to biomodulatory stroma-targeted therapy, since patients with PPARγ-positive metastases showed a significantly prolonged progression-free survival treated with biomodulatory treatment [38]. The expression of PPARγ protein may therefore serve as a positive prognostic marker indicating the responsiveness to stroma-targeted therapy in the metastatic stage (IV) of melanoma. Meyer *et al* suggested that a remodeling of the tumor stroma might be the main target of PPARγ therapy. The recognition of 15d-PGJ2 as a potential anti-tumor drug raises the question of a more detailed understanding of the acting mechanism.

The enhanced knowledge of molecular mechanisms by 15d-PGJ2 generated by shot gun analysis involving important cellular processes, such as cellular signaling networks, regulation of cell cycle, proliferation, transport, cell migration or tumor-stroma interactions may support the design of patient stratification strategies for rational therapeutic concepts.

The data interpretation was supported by the CPL/MUW-database [18]. The number of proteins are automatically classified and provide a fast overview of the main processes involved [18]. Classification considers common household proteins, cell type-specific proteins as well as proteins related to specific functions and enables to decrease the complexicity of data. By comparison of untreated versus treated melanoma cells we were able to confirm the *in vitro* data of the inhibitory effects of 15d-PGJ2 on proliferation, migration and angiogenesis and to extract further relevant proteins involved in tumor progression.

In line with the observation of a decrease of MMP 2 expression in shotgun analysis (downregulation of 1 peptide after 48 hours incubation with 5 µM 15d-PGJ2), we were able to reproduce this downregulation using zymography. This observation supports our argument, that 15d-PGJ2 interferes with the tumor microenvironment.

The identification of less peptides of Hsp90 in 15d-PGJ2-treated A375 compared to untreated cells suggested down-regulation of this protein. Western blot analysis of Hsp90, however, did not support this interpretation. 2D-gel electrophoresis demonstrated a profound change of Hsp protein charge by a pI shift which indicates changes in posttranslational modifications such as phosphorylation.

In addition, western blot analysis showed an upregulation of Hsp56 in 1205Lu. Hsp90 and Hsp56 are known to form complexes playing a role in the intracellular trafficking. Phosphorylation of Hsp56 by CK2 was already demonstrated to influence the formation of the HSP90/HSP56 complex [40]. We propose that the reduction of Hsp90 will lead to an elevation of more unbounded Hsp56.

To strengthen the argument that 15d-PGJ2 might increase Hsp90 phosphorylation and to shed light on the impact of 15d-PGJ2 on the phosphorylation which reflects the activity of the proteins, we performed an IP for phospho-serine followed by shot gun analysis indicating a phosphorylation of several chaperones.

Hsp90 belongs to the best studied molecular chaperones which is required for the stability and function of signaling proteins that promote tumor growth, cell motility and invasion *in vitro* and cancer metastasis *in vivo* [41], [42]. Hsp90 inhibitors exhibit significant anti-neoplastic activity against a broad variety of cancers in preclinical studies, including breast, lung cancer and myeloma as well as melanoma [43], [44]. Thus, blockage of Hsp90 interferes with all anti-cancer mechanisms of 15d-PGJ2 and might be one explanation for the widespread activity of 15d-PGJ2 on tumor progression.

Verification of these considerations will require further investigation of this drug. The present data allow us to conclude that 15d-PGJ2 interferes with several key mechanism of cancer progression [45], since 15d-PGJ2 potently reduced proliferation of melanoma and melanoma-associated cells, induced apoptosis and cell cycle arrest, and diminished tumor migration, lymphangiogenesis and angiogenesis *in vitro*.

In addition we were able to demonstrate the activity of 15d-PGJ2 on melanoma associated fibroblasts. Tumor-associated stroma cells are known to differ from their normal counterparts in the expression of various biologically molecules such as PPARγ [46], which was found to be upregulated in stromal myofibroblasts of colon adenocarcinomas [47].

Two consequences can be deduced from these results: the evaluation of PPARγ expression in tumor stroma and a correlation with features of melanoma patients would be an interesting approach as proposed by Meyer *et al*. and 15d-PGJ2 might serve as an efficient combination therapy with chemotherapeutic agents [29], [38].

The IC50 doses to transfer 15d-PGJ2 as a single compound into an *in vivo* situation are high, but we propose that 15d-PGJ2 might serve as an efficient combination therapy with chemotherapeutic agents by targeting as well the tumor microenvironment.

Our data revealed for the first time a profound effect of 15d-PGJ2 on melanoma cells in addition to the tumor microenvironment suggesting high therapeutic efficiency.

## Materials and Methods

This study was approved by the “ethics committee of the Medical University of Vienna and the general hospital Vienna” (Ethik-Kommission der Medizinischen Universität Wien und des Allgemeinen Krankenhauses der Stadt Wien AKH, EK-Nr.; 093/2003; EK-Nr.: 1088/2009; EK-Nr.: 1123/2009).

### Cell line and Chemicals

M24met cells (kindly provided by Dr. R.A. Reisfeld, Department of Immunology, Scripps Research Institute, La Jolla, CA; [48] were grown in RPMI 1640 supplemented with 10% fetal bovine serum, 2 mM glutamine and 50 µg/ml gentamycin sulfate. The human melanoma cell line 1205Lu isolated of a lung metastasis was cultivated as described previously [49]. A375 and Mel Juso were grown in D-MEM tissue culture medium supplemented with 10% fetal bovine serum, 2 mM glutamine and 50 µg/ml gentamycin sulphate as described previously [50], [51]. Normal human dermal fibroblasts (NHDF) obtained by PromoCell were grown in DMEM (10% FCS). The compounds used in this study were obtained from Eubio (Vienna, Austria) 15d-PGJ2, ciglitazone, troglitazone and WY-14643. All compounds were resolved in DMSO.

### Isolation of melanoma-associated fibroblasts MP9, MP10, MP11 and MCM16

Tumor tissue was digested as described previously [21]. Fibroblasts were magnetically labeled with Anti-Fibroblastic MicroBeads. Cell suspension was loaded onto an MACS Column with a magnetic field. The magnetically labeled fibroblasts were retained within the column and eluted subsequently. Fibroblasts were grown in DMEM (10% FCS). We obtained written informed consent for collecting excised melanocytic lesions of all patients enrolled. This study was approved by the “ethics committee of the Medical University of Vienna and the general hospital Vienna” (Ethik-Kommission der Medizinischen Universität Wien und des Allgemeinen Krankenhauses der Stadt Wien AKH, EK-Nr.; 093/2003; EK-Nr.: 1088/2009).

### Isolation of HUVECs

HUVECs were isolated from umbilical veins and subcultured as described previously [52]. HUVECs were passaged in IMDM (Life Technologies) containing 10% FCS (Life Technologies), streptomycin (100 µg/ml), penicillin (100 U/ml), L-glutamine (2 mM), EC growth supplement with heparin (50 µg/ml; Promocell).

### Isolation of LECs

Neonatal human foreskins were enzymatically digested, the epidermis was removed and dermal cells mechanically released. CD34-positive blood vascular endothelial cells (BVECs) were isolated by immunomagnetic purification with an anti-human CD34 antibody (BD Pharmingen, San Diego, CA) conjugated to immunomagnetic beads (Dynal. Lake Success, NY). The remaining CD34-negative cells were incubated with an immunomagnetic beads-conjugated anti-human CD31 antibody (Dynal) to isolate LECs. LECs were seeded onto fibronectin-coated (1 µl/ml; BD Biosciences, Bedford, MA) culture dishes and propagated in a modified endothelial cell basal medium.

The use of endothelial cells (HUVECs and LECs) has been approved by the “ethics committee of the Medical University of Vienna and the general hospital Vienna” (Ethik-Kommission der Medizinischen Universität Wien und des Allgemeinen Krankenhauses der Stadt Wien AKH, EK-Nr.1123/2009). We obtained written informed consent from all patients (in the case of umbilical cords, written informed consent was obtained from the parents) [53].

Phenotypes of BEC and LEC cultures have been described recently [54] The used LECs are immortalized LECs.

### Cell proliferation-Assay

The CellTiter 96® AQ_ueous_ Non-Radioactive Cell Proliferation Assay (Promega) was used as previously described [21]. In brief, different cell lines or primary cells were plated and treated with increasing concentrations of 15d-PGJ2 or a solvent control. Proliferation was measured at desired time points employing an ELISA plate reader.

### Western Blot

Cells were frozen in liquid nitrogen, lysed and separated by gel electrophoresis as described previously [21], [55]. After blotting membranes were incubated with the following primary antibodies: p21 (1∶200), p53ser37 (1∶200), p53ser15 (1∶200), p53 (1∶200), emmprin (basigin) (1∶200), Mms2 (1∶100), MSH3 (1∶500), Hsp90 (1∶1000), all Santa Cruz Biotechnology, MSH6 (1∶500, Pharmingen) MSH2 (1 µg/ml, Pharmingen), MLH1 (1 µg/ml, Pharmingen) and Nodal (1∶500, Abcam), tubulin (mouse anti-tubulin monoclonal antibody, Sigma Aldrich) or actin (rabbit anti-actin monoclonal antibody, Sigma Aldrich). Binding of primary antibodies was visualized by incubation with horseradish peroxidase conjugated secondary antibodies (anti-mouse IgG or anti-rabbit IgG HRP, both GE Healthcare) followed by chemoluminescent visualization with ECL (Amersham).

### Cell cycle analysis

Cell cycle analysis was performed by propidium iodide FACS staining as described previously [21]. Cells were harvested, and fixed in 70% ethanol RNase (Sigma) was added, cells stained with propidium iodide and analyzed by flow cytometry. Cell cycle distribution was quantified with the ModFIT LT software (Verity Software House, Topsham, ME).

### Matrigel invasion chamber assay

The matrigel invasion chamber assay (BD Biosciences, Bedford, Massachusetts) consists of a two-well chamber system and was peformed as described previously [21]. M24met cells were subjected to different concentrations of 15d-PGJ2 or solvent control. After 48 h, the upper chamber was removed and swiped with a cotton bud. The transmigrated cells on the lower side of the upper chamber were fixed in 70% ethanol and stained using 0.2% crystal blue. Pictures were captured with a AxioCam MRc5 digital camera (Zeiss, Vienna, Austria) attached to an AH3-RFCA microscope (Olympus, Vienna, Austria). The relative amount of transmigrated cells was quantified with a computer-assisted analyses system (Axiovision®)

### Tube formation assay

Matrigel Basement Membrane Matrix (BD) was thawn and 24well plates were coated with 300 µl Matrigel and incubated for 30 minutes at 37°C.

50.000 endothelial cells were seeded and after 8 hours different concentrations of 15d-PGJ2 or solvent control were applied. 24 h after seeding tube formation was documented by the confocal laser microscope (Zeiss).

For Calcein staining 12 or 24 h after seeding, cells were washed once with PBS and cells were incubated for 30 minutes at 37°C with 50 µL PBS containing 0.05% Calcein-AM (Sigma Aldrich, Vienna, Austria). Micrographs of fluorescent cells were taken using a Nikon Digital Sight DS-Fi1C CCD camera. Tube formation was quantified using the Cell Profiler Software Package [56]. Briefly, images were converted into binary images by thresholding. Areas with an extension of more than 125 µm in one direction were considered as tubes and selected for analysis, smaller areas were discarded. A single pixel topological skeleton representing the tubular network was constructed and network length was calculated by multiplying the pixel count with a scaling factor representing microns per pixel.

### Zymography Assay

We stimulated A375 melanoma cells with increasing doses of 15d-PGJ2 (1, 5, 10, 15 µM) for 48 hours. The supernatant was dissolved 1∶1 with MTO-buffer (50 mM Tris, pH 7.5; 200 mM NaCl, 5 mM CaCl_2_) and diluted 1∶1 in sample buffer (100 mM Tris-HCl, pH 6.8, 50% Glycerol, 4% SDS, 0,1% Bromphenolblue). The SDS gel contained gelantine (1 mg/ml). After electrophoresis the gel was incubated with substrate buffer with Triton-X100 (50 mM Tris, pH 7.5; 200 mM NaCl, 5 mM CaCl2, 0,02% Brij; 2.5% Triton-X100) for 1 hour. After incubation substrate buffer without Triton-X100 (50 mM Tris, pH 7.5; 200 mM NaCl, 5 mM CaCl2, 0,02% Brij) at room temperature (2–3 times/hour), the gel was incubated with this buffer over night at 37°C. Subsequently, the gel was stained in Coomassie solution for 30 minutes and stripped with a isopropanol-acetic acid solution (ProteaImmun).

### Proteome Analysis

Shot gun analysis was performed as described previously [17], [39] In brief, cells were fractionated into nuclear, cytoplasmic and secreted protein fractions [57]. Protein fractions were separated by SDS-PAGE, cut into slices and digested with trypsin. Peptides were extracted and separated by nano-flow LC (1100 Series LC system, Agilent, Palo Alto, CA) using the HPLC-Chip technology (Agilent) equipped with a 40 nl Zorbax 300SB-C18 trapping column and a 75 µm×150 mm Zorbax 300SB-C18 separation column at a flow rate of 400 nl/min, using a gradient from 0.2% formic acid and 3% ACN to 0.2% formic acid and 40% ACN over 60 minutes. Peptide identification was accomplished by MS/MS fragmentation analysis with an iontrap mass spectrometer (XCT-Ultra, Agilent) equipped with an orthogonal nanospray ion source. The MS/MS data were interpreted by the Spectrum Mill MS Proteomics Workbench software (Version A.03.03, Agilent) and searched against the SwissProt Database (Version 14.3 containing 20 328 protein entries) allowing for precursor mass deviation of 1.5 Da, a product mass tolerance of 0.7 Da and a minimum matched peak intensity (%SPI) of 70%. Due to previous chemical modification, carbamidomethylation of cysteines was set as fixed modification.

For immunoprecipitation, 5 µg anti-Phosphoserine antibody (PSR-45, Abcam: ab6639) were applied to cytoplasmic protein fractions, followed by an overnight pull-down using Dynal Protein G-coated Dynabeads (Invitrogen). Proteins were released and further processed as described for proteome profiling. In case of the IP analyses, we used a Dionex 3000 nano-LC system and a QEXACTIVE orbitrap mass spectrometer (Thermo). Spectral searches were performed with Mascot.

### 2D- gel electrophoresis

Proteins of A375 melanoma cells treated with 5 µM 15d-PGJ2 or solvent control for 48 hours were loaded by passive rehydration of IPG strips pH 5–8, 17 cm (Bio-Rad, Hercules, CA) at room temperature. IEF was performed in a stepwise fashion (1 h 0–500 V linear; 5 h 500 V; 5 h 500–3500 V linear; 12 h 3500 V). After IEF, the strips were equilibrated with 100 mM DTT and 2.5% iodacetamide according to the instructions of the manufacturer (Bio-Rad Hercules, CA). For SDS-PAGE using the Protean II xi electrophoresis system (Bio-Rad, Hercules, CA, USA), the IPG strips were placed on top of 1.5 mm 12% polyacrylamide slab gels and overlaid with 0.5% low melting agarose. The gels were stained with a 400 nM solution of Ruthenium II tris (bathophenanthroline disulfonate) (RuBPS). Fluorography scanning was performed with the FluorImager 595 (Amersham Biosciences, Amersham, UK) at a resolution of 100 µm. After scanning, gels were dried using the slab gel dryer SE110 (Hoefer, San Francisco CA, USA). Exposure of storage phosphor screens (Molecular Dynamics) occurred at room temperature for 24 h. Screens were subsequently scanned using the Phosphorimager SI (Molecular Dynamics) at a resolution of 100 µm. Proteins were identified by mass spectrometry analysis of tryptic digests of isolated protein spots.

## Supporting Information

Figure S1 **Representative immunoblots of three independent experiments of MMS2, MSH3, MSH6, MSH2, MLH1, Basigin and nodal after 48 hours treatment of 15d-PGJ2 with indicated concentrations (all concentrations are in µM).**
(TIF)

Table S1 **15d-PGJ2 is superior to other PPAR ligands in inhibiting growth of melanoma cell lines, endothelial cells and of tumor associated fibroblasts superior to normal fibroblasts.** Cell viability and proliferation assay. The IC50 is calculated of three independent experiments. IC50 of melanoma cells A375, M24met, 1205Lu, MelJuso and endothelial cells (HUVECs) treated with ciglitazone, troglitazone, 15d-PGJ2 and WY-14643, lymphatic endothelial cells (LECs), normal fibroblasts (NHDF) and tumor-associated fibroblasts treated with 15d-PGJ2.
(PDF)

Table S2 **A, B. Proteins induced by 5 µM 15d-PGJ2 in A375 melanoma cells after 48 hours.** The proteins are classified by the CPL/MUW database. Uniprot serves as reference for the function of the proteins. In addition, the accession numbers are from the Uniprot database. Numbers indicate distinct peptides identified by mass spectrometry. C: cytoplasm, N: nucleous, S: supernatant.
(PDF)

Table S3 **Proteins downregulated by 5 µM 15d-PGJ2 in A375 melanoma cells after 48 hours.** Uniprot serves as reference for the function of the proteins. In addition, the accession numbers are from the Uniprot database. Numbers indicate distinct peptides identified by mass spectrometry.
(PDF)

Table S4 **Chaperones regulated by 5 µM 15d-PGJ2 in A375 melanoma cells after 48 hours.** The accession numbers are from the Uniprot database. Numbers indicate distinct peptides identified by mass spectrometry. C: cytoplasm, N: nucleous, S: supernatant.
(PDF)
