# Supplementary figures and images for: Proteome Analysis Identified the PPARγ Ligand 15d-PGJ2 as a Novel Drug Inhibiting Melanoma Progression and Interfering with Tumor-Stroma Interaction
Source: PLoS One. 2012 Sep 25;7(9):e46103. doi: 10.1371/journal.pone.0046103 (PMC3458105; doi:10.1371/journal.pone.0046103)

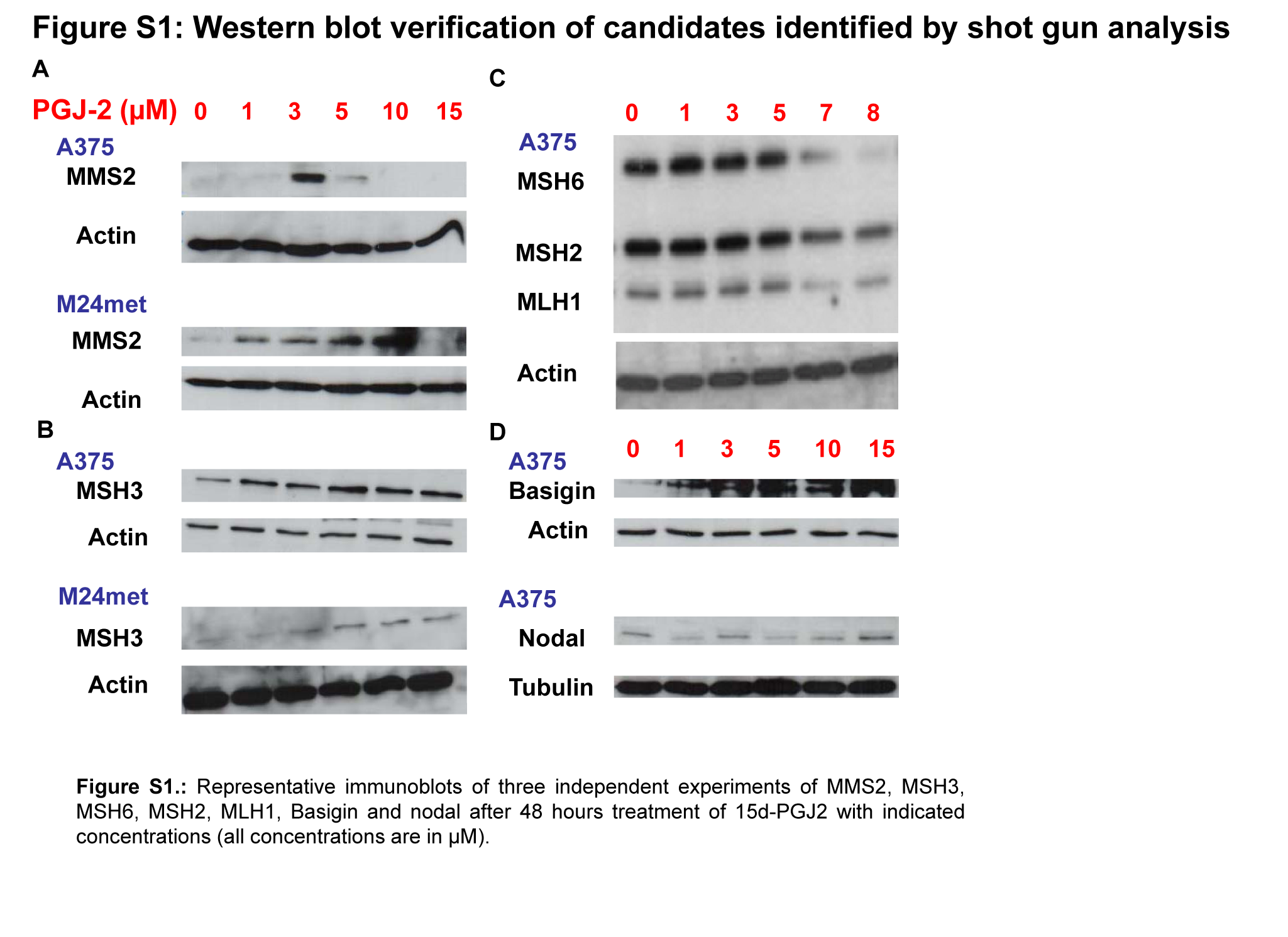

Supplement: Figure S1 — Representative immunoblots of three independent experiments of MMS2, MSH3, MSH6, MSH2, MLH1, Basigin and nodal after 48 hours treatment of 15d-PGJ2 with indicated concentrations (all concentrations are in µM). (TIF) [file pone.0046103.s001.tif]
