# Supplementary material for: Proteome Analysis Identified the PPARγ Ligand 15d-PGJ2 as a Novel Drug Inhibiting Melanoma Progression and Interfering with Tumor-Stroma Interaction
Source: PLoS One. 2012 Sep 25;7(9):e46103. doi: 10.1371/journal.pone.0046103 (PMC3458105; doi:10.1371/journal.pone.0046103)
Supplement: Table S1 — 15d-PGJ2 is superior to other PPAR ligands in inhibiting growth of melanoma cell lines, endothelial cells and of tumor associated fibroblasts superior to normal fibroblasts. Cell viability and proliferation assay. The IC50 is calculated of three independent experiments. IC50 of melanoma cells A375, M24met, 1205Lu, MelJuso and endothelial cells (HUVECs) treated with ciglitazone, troglitazone, 15d-PGJ2 and WY-14643, lymphatic endothelial cells (LECs), normal fibroblasts (NHDF) and tumor-associated fibroblasts treated with 15d-PGJ2. (PDF) [file pone.0046103.s002.pdf]

Table S1: IC50 of cells treated with PPAR ligands

cell line	PPAR ligand	IC50 (µM)
A375 (melanoma cell line)	ciglitazone	>100 (1436)
	troglitazone	>100 (2584)
	15d-PGJ2	**23.4**
	WY-14643	>800 (14394)
M24met	ciglitazone	>100 (2913)
	troglitazone	>100 (1574)
	15d-PGJ2	**25.12**
	WY-14643	674.4
1205Lu	ciglitazone	100.9
	troglitazone	46.09
	15d-PGJ2	**21.97**
	WY-14643	380.9
MelJuso	ciglitazone	>100 (2,721e+007)
	troglitazone	>100 (580.4)
	15d-PGJ2	**37.45**
	WY-14643	791.5
HUVEC (endothelial cells)	ciglitazone	>100 (16242)
	troglitazone	>100 (1615)
	15d-PGJ2	**85.23, 83.7 (2nd isolated cell)**
	WY-14643	>800 (835)
LEC (lymphatic endothelial cells)	15d-PGJ2	**70.84**
**cell line**	**PPAR ligand**	**IC50 (µM)**
NHDF (normal skin fibroblasts)	15d-PGJ2	**127,70**
TF (old)	15d-PGJ2	**92,78**
MP9 fibroblasts	15d-PGJ2	**46,92**
MP10 fibrosblasts	15d-PGJ2	**44,40**
MP11 fibroblasts	15d-PGJ2	**54,40**
MCM16 fibroblasts	15d-PGJ2	**68,22**

Table S1. 15d-PGJ2 is superior to other PPAR ligands in inhibiting growth of melanoma cell lines, endothelial cells and of tumor associated fibroblasts superior to normal fibroblasts. Cell viability and proliferation assay. The IC50 is calculated of three independent experiments. IC50 of melanoma cells A375, M24met, 1205Lu, MelJuso and endothelial cells (HUVECs) treated with ciglitazone, troglitazone, 15d-PGJ2 and WY-14643, lymphatic endothelial cells (LECs), normal fibroblasts (NHDF) and tumor-associated fibroblasts treated with 15d-PGJ2.
