# Supplementary material for: Proteome Analysis Identified the PPARγ Ligand 15d-PGJ2 as a Novel Drug Inhibiting Melanoma Progression and Interfering with Tumor-Stroma Interaction
Source: PLoS One. 2012 Sep 25;7(9):e46103. doi: 10.1371/journal.pone.0046103 (PMC3458105; doi:10.1371/journal.pone.0046103)
Supplement: Table S2 — A, B. Proteins induced by 5 µM 15d-PGJ2 in A375 melanoma cells after 48 hours. The proteins are classified by the CPL/MUW database. Uniprot serves as reference for the function of the proteins. In addition, the accession numbers are from the Uniprot database. Numbers indicate distinct peptides identified by mass spectrometry. C: cytoplasm, N: nucleous, S: supernatant. (PDF) [file pone.0046103.s003.pdf]

# Table S2A: Upregulated candidates by 15d-PGJ2

| Accession | Name | Number of Peptides | Score | Fractions | Function (Uniprot) |
|---|---|---|---|---|---|
| O00506 | Serine/threonine-protein kinase 25 | 1 | 11.78 | C | Oxidant stress-activated serine/threonine kinase that may play a role in the response to environmental stress. Targets to the Golgi apparatus where it appears to regulate protein transport events, cell adhesion, and polarity complexes important for cell migration. Phosphorylated upon DNA damage, probably by ATM or ATR |
| P11166 | Solute carrier family 2, facilitated glucose transporter member 1 | 1 | 16.6 | C | Facilitative glucose transporter. This isoform may be responsible for constitutive or basal glucose uptake. Transport. Phosphorylated upon DNA damage, probably by ATM or ATR. |
| P02647 | Apolipoprotein A-I (Apo-AI) | 1 | 15.85 | C | Participates in the reverse transport of cholesterol from tissues to the liver for excretion by promoting cholesterol efflux from tissues and by acting as a cofactor for the lecithin cholesterol acyltransferase (LCAT). Belongs to the apolipoprotein A1/A4/E family. Transport. Secreted. |
| P61619 | Protein transport protein Sec61 subunit alpha isoform 1 | 2 | 37.67 | C, N | Plays a crucial role in the insertion of secretory and membrane polypeptides into the ER. Required for assembly of membrane and secretory proteins. Transport. |
| O00203 | AP-3 complex subunit beta-1 | 1 | 15.28 | C | Subunit of non-clathrin- and clathrin-associated adaptor protein complex 3 that plays a role in protein sorting in the late-Golgi/trans-Golgi network (TGN) and/or endosomes. The AP complexes mediate both the recruitment of clathrin to membranes and the recognition of sorting signals within the cytosolic tails of transmembrane cargo molecules. AP-3 appears to be involved in the sorting of a subset of transmembrane proteins targeted to lysosomes and lysosome-related organelles. Protein Transport. |
| O00400 | Acetyl-coenzyme A transporter 1 | 1 | 17.26 | C | Probable acetyl-CoA transporter necessary for O-acetylation of gangliosides. Transport |
| O43633 | Charged multivesicular body protein 2a | 1 | 18.49 | C | Probable core component of the endosomal sorting required for transport complex III (ESCRT-III) which is involved in multivesicular bodies (MVBs) formation and sorting of endosomal cargo proteins into MVBs. MVBs are delivered to lysosomes enabling degradation of membrane proteins, such as stimulated growth factor receptors, lysosomal enzymes and lipids. Protein transport. |
| O94979 | Protein transport protein Sec31A | 1 | 16.15 | C | Component of the coat protein complex II (COPII) which promotes the formation of transport vesicles from the endoplasmic reticulum (ER). Protein transport. |
| P03891 | NADH-ubiquinone oxidoreductase chain 2 | 1 | 13.82 | N | Core subunit of the mitochondrial membrane respiratory chain NADH dehydrogenase (Complex I) that is believed to belong to the minimal assembly required for catalysis. Complex I functions in the transfer of electrons from NADH to the respiratory chain. Transport. |
| P07108 | Acyl-CoA-binding protein (ACBP) | 3 | 35.55 | C | Binds medium- and long-chain acyl-CoA esters with very high affinity and may function as an intracellular carrier of acyl-CoA esters. It is also able to displace diazepam from the benzodiazepine (BZD) recognition site located on the GABA type A receptor. Transport. |
| P08574 | Cytochrome c1, heme protein, mitochondrial | 1 | 14.1 | N | This is the heme-containing component of the cytochrome b-c1 complex, which accepts electrons from Rieske protein and transfers electrons to cytochrome c in the mitochondrial respiratory chain. Transport. |

| | | | | | |
|---|---|---|---|---|---|
| P21281 | V-type proton ATPase subunit B, brain isoform | 2 | 30.86 | C | Non-catalytic subunit of the peripheral V1 complex of vacuolar ATPase. V-ATPase is responsible for acidifying a variety of intracellular compartments in eukaryotic cells. Identified by mass spectrometry in melanosome fractions from stage I to stage IV. Transport. |
| P49755 | Transmembrane emp24 domain-containing protein 10 | 1 | 13.61 | C | Involved in vesicular protein trafficking. Identified by mass spectrometry in melanosome fractions from stage I to stage IV. Transport. |
| P53985 | Monocarboxylate transporter 1 (MCT 1) | 1 | 13.46 | C | Proton-linked monocarboxylate transporter. Transport. Widely expressed in normal and in cancer cells. |
| P54709 | Sodium/potassium-transporting ATPase subunit beta-3 (Sodium/potassium-dependent ATPase subunit beta-3) (ATPB-3) | 1 | 14.44 | C | This is the non-catalytic component of the active enzyme, which catalyzes the hydrolysis of ATP coupled with the exchange of Na+ and K+ ions across the plasma membrane. The exact function of the beta-3 subunit is not known. Identified by mass spectrometry in melanosome fractions from stage I to stage IV. Transport. |
| P78363 | Retinal-specific ATP-binding cassette transporter (ATP-binding cassette sub-family A member 4) | 2 | 24.61 | C | May play a role in photoresponse. Retinoids, and most likely retinal, are the natural substrates for transport by abcr in rod outer segments. Belongs to the ABC transporter superfamily. ABCA family. Transport. |
| Q86UQ4 | ATP-binding cassette sub-family A member 13 | 1 | 13.03 | N | Transport |
| Q96CW1 | AP-2 complex subunit mu (Adapter-related protein complex 2 mu subunit) (Adaptor protein complex AP-2 subunit mu) (Adaptin-mu2) (AP-2 mu chain) (Plasma membrane adaptor AP-2 50 kDa protein) (HA2 50 kDa subunit) (Clathrin assembly protein complex 2 medium c | 1 | 12.56 | N | Component of the adaptor protein complex 2 (AP-2). Adaptor protein complexes function in protein transport via transport vesicles in different membrane traffic pathways. Adaptor protein complexes are vesicle coat components and appear to be involved in cargo selection and vesicle formation. AP-2 is involved in clathrin-dependent endocytosis in which cargo proteins are incorporated into vesicles surrrounded by clathrin (clathrin-coated vesicles, CCVs) which are destined for fusion with the early endosome. |
| Q96M27 | Protein PRRC1 | 1 | 15.1 | C | Golgi apparatus |
| Q9UKS6 | Protein kinase C and casein kinase substrate in neurons protein 3 | 1 | 13.92 | N | May play a role in vesicle formation and transport |
| Q9Y5W7 | Sorting nexin-14 | 1 | 14.65 | C | May be involved in several stages of intracellular trafficking. Transport. |
| O60231 | Putative pre-mRNA-splicing factor ATP-dependent RNA helicase DHX16 | 1 | 17.61 | N | Probable ATP-binding RNA helicase involved in pre-mRNA splicing. |
| O60306 | Intron-binding protein aquarius (Intron-binding protein of 160 kDa) (IBP160) | 3 | 31.31 | N | Intron-binding spliceosomal protein required to link pre-mRNA splicing and snoRNP (small nucleolar ribonucleoprotein) biogenesis. |
| Q16560 | U11/U12 small nuclear ribonucleoprotein 35 kDa protein | 1 | 17.13 | N | Component of the U11/U12 snRNPs that are part of the U12-type spliceosome. |
| Q6PIY7 | Poly(A) RNA polymerase GLD2 (hGLD-2) (PAP-associated domain-containing protein 4) (Terminal uridylyltransferase 2) (TUTase 2) | 1 | 15.71 | N | Cytoplasmic poly(A) RNA polymerase that adds successive AMP monomers to the 3'-end of specific RNAs, forming a poly(A) tail. In contrast to the canonical nuclear poly(A) RNA polymerase, it only adds poly(A) to selected cytoplasmic mRNAs. Does not play a role in replication-dependent histone mRNA degradation |
| Q96I25 | Splicing factor 45 (45 kDa-splicing factor) (RNA-binding motif protein 17) | 1 | 13.29 | N | Splice factor that binds to the single stranded 3'AG at the exon/intron border and promotes its utilization in the second catalytic step. Involved in the regulation of alternative splicing and the utilization of cryptic splice sites. Promotes the utilization of a cryptic splice site created by the beta-110 mutation in the HBB gene. The resulting frameshift leads to sickle cell anemia |
| P63162 | Small nuclear ribonucleoprotein-associated protein N | 7 | 105.3 | N | May be involved in tissue-specific alternative RNA processing events. |
| Q12799 | T-complex protein 10A homolog | 1 | 11.85 | C | Alternative Splicing. |

| | | | | | |
|---|---|---|---|---|---|
| Q16637 | Survival motor neuron protein (Component of gems 1) (Gemin-1) | 1 | 13.83 | C | The SMN complex plays an essential role in spliceosomal snRNP assembly in the cytoplasm and is required for pre-mRNA splicing in the nucleus. It may also play a role in the metabolism of snoRNPs |
| Q3KQU3 | MAP7 domain-containing protein 1 | 1 | 15.84 | C | Alternative Splicing. |
| Q5T3F8 | Transmembrane protein 63B | 1 | 19.77 | N | Membrane. Alternative Splicing. |
| Q5T8P6 | RNA-binding protein 26 | 1 | 16.07 | N | Alternative Splicing. |
| Q5TB30 | DEP domain-containing protein 1A | 1 | 12.2 | C | Alternative Splicing. Up-regulated in bladder cancer cells (at protein level). |
| Q96D71 | RalBP1-associated Eps domain-containing protein 1 | 1 | 13.78 | C | May coordinate the cellular actions of activated EGF receptors and Ral-GTPases |
| Q14004 | Cell division protein kinase 13 (Cell division cycle 2-like protein kinase 5) (CDC2-related protein kinase 5) | 1 | 17.17 | N | May be a controller of the mitotic cell cycle. Involved in the blood cell development. Also expressed in neuroblastoma and glioblastoma tumors. Phosphorylated upon DNA damage, probably by ATM or ATR. |
| Q14839 | Chromodomain-helicase-DNA-binding protein 4 | 1 | 12.43 | N | Probable transcription regulator. Phosphorylated upon DNA damage, probably by ATM or ATR. |
| Q99575 | Ribonucleases P/MRP protein subunit POP1 (hPOP1) | 2 | 29.55 | N | Component of ribonuclease P, a protein complex that generates mature tRNA molecules by cleaving their 5'-ends. Also a component of RNase MRP Phosphorylated upon DNA damage, probably by ATM or ATR |
| Q9C0C2 | 182 kDa tankyrase-1-binding protein | 4 | 54.84 | C | Binds to the ANK repeat domain of TNKS1 and TNKS2.Phosphorylated upon DNA damage, probably by ATM or ATR |
| O60271 | C-jun-amino-terminal kinase-interacting protein 4 (JNK-interacting protein 4) | 1 | 16.37 | C | The JNK-interacting protein (JIP) group of scaffold proteins selectively mediates JNK signaling by aggregating specific components of the MAPK cascade to form a functional JNK signaling module. Perinuclear distribution in response to stress signals such as UV radiation. Phosphorylated upon DNA damage, probably by ATM or ATR. |
| P20585 | DNA mismatch repair protein Msh3 (hMSH3) (Divergent upstream protein) (DUP) (Mismatch repair protein 1) (MRP1) | 2 | 19.2 | N | Component of the post-replicative DNA mismatch repair system (MMR). Heterodimerizes with MSH2 to form MutS beta which binds to DNA mismatches thereby initiating DNA repair. Phosphorylated upon DNA damage, probably by ATM or ATR. |
| Q02952 | A-kinase anchor protein 12 (A-kinase anchor protein 250 kDa) (AKAP 250) (Gravin) (Myasthenia gravis autoantigen) | 1 | 18.9 | C | Anchoring protein that mediates the subcellular compartmentation of protein kinase A (PKA) and protein kinase C (PKC). Expressed in endothelial cells, cultured fibroblasts and osteosarcoma. Activated by lysophosphatidylcholine (lysoPC). Phosphorylated upon DNA damage, probably by ATM or ATR. |
| Q15554 | Telomeric repeat-binding factor 2 (TTAGGG repeat-binding factor 2) (Telomeric DNA-binding protein) | 1 | 14.27 | N | Binds the telomeric double-stranded TTAGGG repeat. Protects against end-to-end fusion of chromosomes and plays a role in successful progression through the cell division cycle. Component of the shelterin complex (telosome) that is involved in the regulation of telomere length and protection. Phosphorylated upon DNA damage, probably by ATM or ATR.Shelterin associates with arrays of double-stranded TTAGGG repeats added by telomerase and protects chromosome ends; without its protective activity, telomeres are no longer hidden from the DNA damage surveillance and chromosome ends are inappropriately processed by DNA repair pathways. |
| Q9H583 | HEAT repeat-containing protein 1 (Protein BAP28) | 3 | 36.74 | N | Involved in nucleolar processing of pre-18S ribosomal RNA. Involved in ribosome biosynthesis Phosphorylated upon DNA damage, probably by ATM or ATR. |

| | | | | | |
|---|---|---|---|---|---|
| Q9NPQ8 | Synembryn-A (Protein Ric-8A) | 2 | 29.98 | C | Guanine nucleotide exchange factor (GEF), which can activate some, but not all, G-alpha proteins. Able to activate GNAI1, GNAO1 and GNAQ, but not GNAS by exchanging bound GDP for free GTP. Involved in regulation of microtubule pulling forces during mitotic movement of chromosomes by stimulating G(i)-alpha protein, possibly leading to release G(i)-alpha-GTP and NuMA proteins from the NuMA-GPSM2-G(i)-alpha-GDP complex By similarity. Also acts as an activator for G(q)-alpha (GNAQ) protein by enhancing the G(q)-coupled receptor-mediated ERK activation Phosphorylated upon DNA damage, probably by ATM or ATR |
| Q9UNF1 | Melanoma-associated antigen D2 (MAGE-D2 antigen) (Breast cancer-associated gene 1 protein) (BCG-1) (11B6) (Hepatocellular carcinoma-associated protein JCL-1) | 1 | 9.3 | C | Phosphorylated upon DNA damage, probably by ATM or ATR |
| P24557 | Thromboxane-A synthase (TXA synthase) (Cytochrome P450 5A1) | 1 | 12.57 | C | Belongs to the cytochrome P450 family. Fatty acid biosynthesis, Lipid synthesis, Prostaglandin biosynthesis |
| P30837 | Aldehyde dehydrogenase X, mitochondrial (Aldehyde dehydrogenase family 1 member B1) (Aldehyde dehydrogenase 5) | 1 | 12.33 | C | ALDHs play a major role in the detoxification of alcohol-derived acetaldehyde. They are involved in the metabolism of corticosteroids, biogenic amines, neurotransmitters, and lipid peroxidation. |
| P54619 | 5'-AMP-activated protein kinase subunit gamma-1 (AMPK subunit gamma-1) | 1 | 21.4 | C | AMPK is responsible for the regulation of fatty acid synthesis by phosphorylation of acetyl-CoA carboxylase. Also regulates cholesterol synthesis via phosphorylation and inactivation of hydroxymethylglutaryl-CoA reductase and hormone-sensitive lipase. Fatty acid biosynthesis, Lipid synthesis |
| Q14914 | Prostaglandin reductase 1 (PRG-1) (NADP-dependent leukotriene B4 12-hydroxydehydrogenase) (15-oxoprostaglandin 13-reductase) | 1 | 13.61 | C | Functions as 15-oxo-prostaglandin 13-reductase and acts on 15-oxo-PGE1, 15-oxo-PGE2 and 15-oxo-PGE2-alpha. Has no activity towards PGE1, PGE2 and PGE2-alpha By similarity. Catalyzes the conversion of leukotriene B4 into its biologically less active metabolite, 12-oxo-leukotriene B4. This is an initial and key step of metabolic inactivation of leukotriene B4. |
| Q96Q06 | Perilipin-4 (Adipocyte protein S3-12) | 1 | 16.51 | C | May play a role in triacylglycerol packaging into adipocytes. May function as a coat protein involved in the biogenesis of lipid droplets Up-regulated during adipocyte differentiation |
| Q99541 | Adipophilin (Adipose differentiation-related protein) (ADRP) | 1 | 13.13 | C | May be involved in development and maintenance of adipose tissue |
| P35659 | Protein DEK | 3 | 47.66 | N | May have a function in the nucleus. DEK is found in a subset of acute myeloid leukemia (AML); also known as acute non-lymphocytic leukemia. |
| P42695 | Condensin-2 complex subunit D3 (Non-SMC condensin II complex subunit D3) (hCAP-D3) | 1 | 12.79 | N | Regulatory subunit of the condensin-2 complex, a complex which establishes mitotic chromosome architecture and is involved in physical rigidity of the chromatid axis. Cell cycle, cell division, mitosis. |
| Q12974 | Protein tyrosine phosphatase type IVA 2 | 1 | 17.79 | C | Protein tyrosine phosphatase which stimulates progression from G1 into S phase during mitosis. Promotes tumors. Overexpressed in prostate tumor tissue. |
| Q15155 | Nodal modulator 1 (pM5) | 1 | 14.64 | N | May antagonize Nodal signaling . Expressed in colon tumor tissue and in adjacent normal colonic mucosa. Pro-apoptotic, Anti-proliferation. |
| Q15287 | RNA-binding protein with serine-rich domain 1 (SR-related protein LDC2) | 1 | 17.31 | N | Component of a splicing-dependent multiprotein exon junction complex (EJC) deposited at splice junction on mRNAs. |
| Q15477 | Helicase SKI2W (Helicase-like protein) (HLP) | 1 | 17.15 | C | Helicase; has ATPase activity. |
| Q16527 | Cysteine and glycine-rich protein 2 (Cysteine-rich protein 2) (Smooth muscle cell LIM protein) | 1 | 12.26 | N | Drastically down-regulated in response to PDGF-BB or cell injury, that promote smooth muscle cell proliferation and dedifferentiation. Seems to play a role in the development of the embryonic vascular system. |

| | | | | | |
|---|---|---|---|---|---|
| Q7KZ85 | Transcription elongation factor SPT6 (hSPT6) | 1 | 18.7 | N | Acts to stimulate transcriptional elongation by RNA polymerase II |
| Q7L2E3 | Putative ATP-dependent RNA helicase DHX30 | 2 | 24.85 | N | no known function |
| Q7Z5K2 | Wings apart-like protein homolog (Friend of EBNA2 protein) | 1 | 14.39 | N | May play a role in cell growth. |
| Q8NBU5 | ATPase family AAA domain-containing protein 1 | 1 | 17.16 | N | Replication |
| Q8TF68 | Zinc finger protein 384 | 1 | 13.86 | N | Transcription factor that binds the consensus DNA sequence [GC]AAAAA. Seems to bind and regulate the promoters of MMP1, MMP3, MMP7 and COL1A1 |
| Q969S3 | Zinc finger protein 622 (Zinc finger-like protein 9) | 1 | 15.58 | N | May behave as an activator of the bound transcription factor, MYBL2, and be involved in embryonic development |
| Q96RK0 | Protein capicua homolog | 1 | 12.71 | C | Transcriptional repressor which may play a role in development of the central nervous system (CNS) |
| Q9H6F5 | Coiled-coil domain-containing protein 86 | 1 | 14.65 | N | Nuclear |
| Q9Y5V0 | Zinc finger protein 706 | 1 | 13.22 | N | Zinc finger protein. |
| O00754 | Lysosomal alpha-mannosidase (Laman) | 1 | 14.91 | C | Necessary for the catabolism of N-linked carbohydrates released during glycoprotein turnover. Cleaves all known types of alpha-mannosidic linkages. |
| O14880 | Microsomal glutathione S-transferase 3 | 1 | 15.48 | N | Functions as a glutathione peroxidase. |
| O15143 | Actin-related protein 2/3 complex subunit 1B (Arp2/3 complex 41 kDa subunit) (p41-ARC) | 1 | 15.84 | C | Functions as component of the Arp2/3 complex which is involved in regulation of actin polymerization and together with an activating nucleation-promoting factor (NPF) mediates the formation of branched actin networks. |
| O15511 | Actin-related protein 2/3 complex subunit 5 (Arp2/3 complex 16 kDa subunit) (p16-ARC) | 1 | 13.18 | C | Functions as component of the Arp2/3 complex which is involved in regulation of actin polymerization and together with an activating nucleation-promoting factor (NPF) mediates the formation of branched actin networks. |
| O43516 | WAS/WASL-interacting protein family member 1 | 1 | 12.63 | C | May have direct activity on the actin cytoskeleton. Induces actin polymerization and redistribution. |
| O75179 | Ankyrin repeat domain-containing protein 17 | 1 | 15.62 | C | Earliest specific in situ marker of hepatic differentiation during embryogenesis, useful for characterization of inductive events involved in hepatic specification |
| O75293 | Growth arrest and DNA damage-inducible protein GADD45 beta | 1 | 11.3 | C | Involved in the regulation of growth and apoptosis. Mediates activation of stress-responsive MTK1/MEKK4 MAPKKK. |
| O76039 | Cyclin-dependent kinase-like 5 (Serine/threonine-protein kinase 9) | 1 | 12.29 | N | Mediates phosphorylation of MECP2 |
| O94804 | Serine/threonine-protein kinase 10 (Lymphocyte-oriented kinase) | 1 | 14.73 | C | Can act on substrates such as myelin basic protein and histone 2A on serine and threonine residues |
| O95139 | NADH dehydrogenase [ubiquinone] 1 beta subcomplex subunit 6 (NADH-ubiquinone oxidoreductase B17 subunit) (Complex I-B17) (CI-B17) | 1 | 12.64 | N | Accessory subunit of the mitochondrial membrane respiratory chain NADH dehydrogenase (Complex I), that is believed not to be involved in catalysis. Complex I functions in the transfer of electrons from NADH to the respiratory chain. The immediate electron acceptor for the enzyme is believed to be ubiquinone. |
| P05362 | Intercellular adhesion molecule 1 (ICAM-1) (Major group rhinovirus receptor) | 1 | 12.01 | C | ICAM proteins are ligands for the leukocyte adhesion protein LFA-1 (integrin alpha-L/beta-2). During leukocyte trans-endothelial migration, ICAM1 engagement promotes the assembly of endothelial apical cups through SGEF and RHOG activation. |
| P09601 | Heme oxygenase 1 (HO-1) | 2 | 19.3 | C | Heme oxygenase cleaves the heme ring at the alpha methene bridge to form biliverdin. Biliverdin is subsequently converted to bilirubin by biliverdin reductase. |
| P10636 | Microtubule-associated protein tau (Neurofibrillary tangle protein) (Paired helical filament-tau) (PHF-tau) | 1 | 13.28 | N | Promotes microtubule assembly and stability, and might be involved in the establishment and maintenance of neuronal polarity. The C-terminus binds axonal microtubules while the N-terminus binds neural plasma membrane components, suggesting that tau functions as a linker protein between both. |

| | | | | | |
|---|---|---|---|---|---|
| P11279 | Lysosome-associated membrane glycoprotein 1 (LAMP-1) (CD107 antigen-like family member A) | 1 | 15.01 | C | Presents carbohydrate ligands to selectins. Also implicated in tumor cell metastasis. |
| P11474 | Steroid hormone receptor ERR1 (Estrogen-related receptor alpha) (ERR-alpha) (Estrogen receptor-like 1) (Nuclear receptor subfamily 3 group B member 1) | 1 | 13.81 | N | Belongs to the nuclear hormone receptor family. Binds to an ERR-alpha response element (ERRE) containing a single consensus half-site. Binds DNA as a monomer or a homodimer. |
| P16615 | Sarcoplasmic/endoplasmic reticulum calcium ATPase 2 | 3 | 44.84 | C, N | This magnesium-dependent enzyme catalyzes the hydrolysis of ATP coupled with the translocation of calcium from the cytosol to the sarcoplasmic reticulum lumen. |
| P16930 | Fumarylacetoacetase (FAA) | 1 | 12.36 | C | 4-fumarylacetoacetate + H2O = acetoacetate + fumarate. |
| P17275 | Transcription factor jun-B | 1 | 12.94 | N | Transcription factor involved in regulating gene activity following the primary growth factor response. Binds to the DNA sequence 5'-TGA[CG]TCA-3'.Transcription. |
| P17661 | Desmin | 1 | 12.85 | N | Desmin are class-III intermediate filaments found in muscle cells. Belongs to the intermediate filament family. |
| P27105 | Erythrocyte band 7 integral membrane protein (Stomatin) | 2 | 27.52 | C, N | Thought to regulate cation conductance. Identified by mass spectrometry in melanosome fractions. |
| P29144 | Tripeptidyl-peptidase 2 | 1 | 13.67 | C | Component of the proteolytic cascade acting downstream of the 26S proteasome in the ubiquitin-proteasome pathway. |
| P30043 | Flavin reductase (FR) (NADPH-dependent diaphorase) | 1 | 15.75 | C | Broad specificity oxidoreductase that catalyzes the NADPH-dependent reduction of a variety of flavins, such as riboflavin, FAD or FMN, biliverdins, methemoglobin and PQQ (pyrroloquinoline quinone). Contributes to heme catabolism and metabolizes linear tetrapyrroles |
| P35613 | Basigin (Leukocyte activation antigen M6) (Collagenase stimulatory factor) | 2 | 26.82 | C | Plays pivotal roles in spermatogenesis, embryo implantation, neural network formation and tumor progression. Stimulates adjacent fibroblasts to produce matrix metalloproteinases (MMPS). Seems to be a receptor for oligomannosidic glycans. In vitro, promotes outgrowth of astrocytic processes .Enriched on the surface of tumor cells. Up-regulated in gliomas. Its expression levels correlate with malignant potential of the tumor. |
| P36957 | Dihydrolipoyllysine-residue succinyltransferase component of 2-oxoglutarate dehydrogenase complex, mitochondrial | 1 | 19.07 | N | The 2-oxoglutarate dehydrogenase complex catalyzes the overall conversion of 2-oxoglutarate to succinyl-CoA and CO2. It contains multiple copies of 3 enzymatic components: 2-oxoglutarate dehydrogenase (E1), dihydrolipoamide succinyltransferase (E2) and lipoamide dehydrogenase (E3). Mitochondrial. |
| P40121 | Macrophage-capping protein (Actin regulatory protein CAP-G) | 1 | 12.89 | C | Calcium-sensitive protein which reversibly blocks the barbed ends of actin filaments but does not sever preformed actin filaments. May play an important role in macrophage function. May play a role in regulating cytoplasmic and/or nuclear structures through potential interactions with actin. May bind DNA. Identified by mass spectrometry in melanosome fractions from stage I to stage IV. |
| P48163 | NADP-dependent malic enzyme (NADP-ME) (Malic enzyme 1) | 1 | 14.28 | C | (S)-malate + NADP+ = pyruvate + CO2 + NADPH. Oxidoreductase |
| P49662 | Caspase-4 (CASP-4) (Protease ICH-2) (Protease TX) (ICE(rel)-II) | 1 | 13.83 | C | Involved in the activation cascade of caspases responsible for apoptosis execution. Cleaves caspase-1. |
| P55786 | Puromycin-sensitive aminopeptidase (PSA) | 4 | 52.95 | C | Aminopeptidase with broad substrate specificity to several peptides. Involved in proteolytic events essential for cell growth and viability. Identified by mass spectrometry in melanosome fractions from stage I to stage IV. |
| P57088 | Transmembrane protein 33 (Protein DB83) | 1 | 17.38 | C, N | Transmembrane. Identified by mass spectrometry in melanosome fractions from stage I to stage IV. |
| Q14139 | Ubiquitin conjugation factor E4 A | 1 | 11.97 | C | Binds to the ubiquitin moieties of preformed conjugates and catalyzes ubiquitin chain assembly in conjunction with E1, E2, and E3. mRNA processing |
| Q14147 | Probable ATP-dependent RNA helicase DHX34 | 1 | 12.57 | N | Probable ATP-binding RNA helicase. |

| | | | | | |
|---|---|---|---|---|---|
| Q15819 | Ubiquitin-conjugating enzyme E2 variant 2 (MMS2) (Enterocyte differentiation-associated factor EDAF-1) (Enterocyte differentiation-promoting factor) (EDPF-1) (Vitamin D3-inducible protein) (DDVit 1) | 2 | 24.62 | C | Has no ubiquitin ligase activity on its own. The UBE2V2/UBE2N heterodimer catalyzes the synthesis of non-canonical poly-ubiquitin chains that are linked through 'Lys-63'. This type of poly-ubiquitination does not lead to protein degradation by the proteasome. Mediates transcriptional activation of target genes. Plays a role in the control of progress through the cell cycle and differentiation. Plays a role in the error-free DNA repair pathway and contributes to the survival of cells after DNA damage. |
| Q16719 | Kynureninase | 3 | 40.75 | C | Pyridine nucleotide biosynthesis |
| Q5T655 | Coiled-coil domain-containing protein 147 | 1 | 14.65 | C | Not known. |
| Q6WCQ1 | Myosin phosphatase Rho-interacting protein (M-RIP) (Rho-interacting protein 3) (RIP3) (p116Rip) | 1 | 14.68 | N | Targets myosin phosphatase to the actin cytoskeleton. Required for the regulation of the actin cytoskeleton by RhoA and ROCK1. Depletion leads to an increased number of stress fibers in smooth muscle cells through stabilization of actin fibers by phosphorylated myosin. Overexpression of MRIP as well as its F-actin-binding region leads to disassembly of stress fibers in neuronal cells |
| Q6ZS46 | Putative uncharacterized protein FLJ45840 | 1 | 12.15 | N | no known function |
| Q7Z2T5 | TRM1-like protein | 1 | 13.84 | N | May play a role in motor coordination and exploratory behavior |
| Q7Z6I8 | UPF0461 protein C5orf24 | 1 | 13.57 | N | no known function |
| Q8IW41 | MAP kinase-activated protein kinase 5 | 1 | 14.63 | C | Mediates stress-induced small heat shock protein 27 phosphorylation |
| Q8IY37 | Probable ATP-dependent RNA helicase DHX37 (DEAH box protein 37) | 2 | 29.56 | N | Belongs to the DEAD box helicase family. DEAH subfamily. |
| Q8IYD1 | Eukaryotic peptide chain release factor GTP-binding subunit ERF3B (Eukaryotic peptide chain release factor subunit 3b) (G1 to S phase transition protein 2 homolog) | 2 | 27.01 | C | Involved in translation termination in response to the termination codons UAA, UAG and UGA. May play a role as a potent stimulator of the release factor activity Highly expressed in IUCC stage II colorectal cancer (CRC) of ETF1. Exhibits GTPase activity, which is ribosome- and ETF1-dependent. May play a role in cell cycle progression. |
| Q8N6L1 | Keratinocyte-associated protein 2 (KCP-2) | 1 | 13.2 | N | Component of the oligosaccharyltransferase (OST) complex. OST seems to exist in different forms which contain at least RPN1, RPN2, OST48, DAD1, OSTC, KRTCAP2 and either STT3A or STT3B. OST can form stable complexes with the Sec61 complex or with both the Sec61 and TRAP complexes |
| Q8N766 | Uncharacterized protein KIAA0090 | 1 | 13.36 | N | no known function |
| Q8NA47 | Coiled-coil domain-containing protein 63 | 1 | 13.18 | N | no known function |
| Q8NEF9 | Serum response factor-binding protein 1 (SRF-dependent transcription regulation-associated protein) (p49/STRAP) | 1 | 13.53 | N | May be involved in regulating transcriptional activation of cardiac genes during the aging process. May play a role in biosynthesis and/or processing of SLC2A4 in adipose cells |
| Q8TBC4 | NEDD8-activating enzyme E1 catalytic subunit (Ubiquitin-like modifier-activating enzyme 3) (Ubiquitin-activating enzyme 3) (NEDD8-activating enzyme E1C) (Ubiquitin-activating enzyme E1C) | 1 | 14.4 | C | Catalytic subunit of the dimeric UBA3-NAE1 E1 enzyme. E1 activates NEDD8 by first adenylating its C-terminal glycine residue with ATP, thereafter linking this residue to the side chain of the catalytic cysteine, yielding a NEDD8-UBA3 thioester and free AMP. E1 finally transfers NEDD8 to the catalytic cysteine of UBE2M. Down-regulates steroid receptor activity. Necessary for cell cycle progression. |
| Q96KB5 | Lymphokine-activated killer T-cell-originated protein kinase | 1 | 14.29 | C | Phosphorylates MAP kinase p38. Seems to be active only in mitosis. May also play a role in the activation of lymphoid cells. When phosphorylated, forms a complex with TP53, leading to TP53 destabilization and attenuation of G2/M checkpoint during doxorubicin-induced DNA damage |
| Q96N11 | Uncharacterized protein C7orf26 | 1 | 11.54 | N | no known function |

| | | | | | |
|---|---|---|---|---|---|
| Q9BQ52 | Zinc phosphodiesterase ELAC protein 2 | 1 | 13.44 | C | Zinc phosphodiesterase, which displays some tRNA 3'-processing endonuclease activity. Probably involved in tRNA maturation, by removing a 3'-trailer from precursor tRNA. |
| Q9BV86 | Methyltransferase-like protein 11A | 1 | 12.02 | C | Probable S-adenosyl-L-methionine-dependent methyltransferase |
| Q9BZ29 | Dedicator of cytokinesis protein 9 (Cdc42 guanine nucleotide exchange factor zizimin-1) | 1 | 12.47 | C | Guanine nucleotide-exchange factor (GEF) that activates CDC42 by exchanging bound GDP for free GTP. Overexpression induces filopodia formation |
| Q9GZS1 | DNA-directed RNA polymerase I subunit RPA49 (RNA polymerase I subunit A49) (DNA-directed RNA polymerase I subunit E) (RNA polymerase I-associated factor 1) (RNA polymerase I-associated factor 53) | 1 | 12.69 | N | DNA-dependent RNA polymerase catalyzes the transcription of DNA into RNA using the four ribonucleoside triphosphates as substrates. Component of RNA polymerase I which synthesizes ribosomal RNA precursors. Appears to be involved in the formation of the initiation complex at the promoter by mediating the interaction between Pol I and UBTF/UBF |
| Q9H501 | ESF1 homolog (ABT1-associated protein) | 1 | 12.72 | N | May constitute a novel regulatory system for basal transcription. Negatively regulates ABT1 |
| Q9H853 | Putative tubulin-like protein alpha-4B (Alpha-tubulin 4B) | 2 | 23.58 | N | cytoskeleton |
| Q9NSD9 | Phenylalanyl-tRNA synthetase beta chain (Phenylalanine--tRNA ligase beta chain) (PheRS) | 3 | 44.85 | N | ATM target |
| Q9NUQ9 | Protein FAM49B (L1) | 1 | 13.07 | C | no known function |
| Q9NX40 | OCIA domain-containing protein 1 | 3 | 41.16 | C, N | no known function |
| Q9NZA1 | Chloride intracellular channel protein 5 | 1 | 13 | C | Can insert into membranes and form poorly selective ion channels that may also transport chloride ions. May play a role in the regulation of transepithelial ion absorption and secretion. Required for normal formation of stereocilia in the inner ear and normal development of the organ of Corti |
| Q9P000 | COMM domain-containing protein 9 | 1 | 16.62 | C | no known function |
| Q9P2D3 | HEAT repeat-containing protein 5B | 1 | 15.1 | C | no known function |
| Q9UET6 | Putative ribosomal RNA methyltransferase 1 | 1 | 14.55 | C | S-adenosyl-L-methionine + rRNA = S-adenosyl-L-homocysteine + rRNA containing 2'-O-methyluridine. |
| Q9UJY1 | Heat shock protein beta-8 (HspB8) | 1 | 13.43 | C | Displays temperature-dependent chaperone activity. |
| Q9UM00 | Transmembrane and coiled-coil domain-containing protein 1 | 1 | 13.21 | N | Endoplasmic reticulum membrane; |
| Q9UM54 | Myosin-VI (Unconventional myosin VI) | 2 | 26.36 | C | Transport |
| Q9Y263 | Phospholipase A-2-activating protein (PLA2P) | 1 | 15.17 | C | Plays an important role in the regulation of specific inflammatory disease processes |
| Q9Y2R0 | Coiled-coil domain-containing protein 56 | 1 | 14.32 | C | no known function |
| Q9Y399 | 28S ribosomal protein S2, mitochondrial (S2mt) | 1 | 14.43 | C | Component of the mitochondrial ribosome small subunit (28S) . |
| Q9Y5T4 | DnaJ homolog subfamily C member 15 (Methylation-controlled J protein) (MCJ) (Cell growth-inhibiting gene 22 protein) | 1 | 17 | N | Absent or down-regulated in many advanced cases of ovarian adenocarcinoma, due to hypermethylation and allelic loss. Loss expression correlates with increased resistance to antineoplastic drugs, such as cisplatin. |

# Table S2B: Categories of upregulated candidates by 15d-PGJ2

| Accession | Transport | mRNA processing | ATM target | Lipid metabolism | Nuclear | cell cycle | Cell Stress | Ciplatin-R | Cytoskeleton | Detoxification | DNA damage/repair | Drug resistance | ECM | Genuinely Secreted | Inflammation | Lysosome | Membrane | Migration | Mitochondrial | Pro-apoptotic | Proteases/Inhibitors | Protein degradation | Protein synthesis | Redox | Replication | Ribosomal | signalling | Transcription | Activation | Anti-apoptotic |
|---|---|---|---|---|---|---|---|---|---|---|---|---|---|---|---|---|---|---|---|---|---|---|---|---|---|---|---|---|---|---|
| O00506 | x | | x | | | | x | | | | | | | | | | | x | | | | | | | | | x | | | |
| P11166 | x | | x | | | | | | | | | | | | | | | | | | | | | | | | | | | |
| P02647 | x | | | x | | | | | | | | | | x | | | | | | | | | | | | | | | | |
| P61619 | x | | | | x | | | x | | | | | | | | | | | | | | | | | | | | | | |
| O00203 | x | | | | | | | | | | | | | | | x | | | | | | | | | | | | | | |
| O00400 | x | | | | | | | | | | | | | | | | | | | | | | | | | | | | | |
| O43633 | x | | | | | | | | | | | | | | | x | | | | | | | | | | | | | | |
| O94979 | x | | | | | | | | | | | | | | | | | | | | | | | | | | | | | |
| P03891 | x | | | | | | | | | | | | | | | | | | x | | | | | | | | | | | |
| P07108 | x | | | | | | | | | | | | | | | | | | | | | | | | | | | | | |
| P08574 | x | | | | | | | x | | | | | | | | | | | x | | | | | | | | | | | |
| P21281 | x | | | | | | | | | | | | | | | | | | | | | | | | | | | | | |
| P49755 | x | | | | | | | | | | | | | | | | x | | | | | | | | | | | | | |
| P53985 | x | | | | | | | | | | | | | | | | | | | | | | | | | | | | | |
| P54709 | x | | | | | | | | | | | | | | | | | | | | | | | | | | | | | |
| P78363 | x | | | | | | | | | x | | | | | | | | | | | | | | | | | | | | |
| Q86UQ4 | x | | | | | | | | | | | | | | | | | | | | | | | | | | | | | |
| Q96CW1 | x | | | | | | | | | | | | | | | | | | | | | | | | | | | | | |
| Q96M27 | x | | | | | | | | | | | | | | | | | | | | | | | | | | | | | |
| Q9UKS6 | x | | | | | | | | | | | | | | | | | | | | | | | | | | | | x | |
| Q9Y5W7 | x | | | | | | | | | | | | | | | | | | | | | | | | | | | | | |
| O60231 | | x | | | x | | | | | | | | | | | | | | | | | | | | | | | | | |
| O60306 | | x | | | x | | | | | | | | | | | | | | | | | | | | | | | | | |
| Q16560 | | x | | | x | | | | | | | | | | | | | | | | | | | | | | | | | |
| Q6PIY7 | | x | | | x | | | | | | | | | | | | | | | | | | | | | | | | | |
| Q96I25 | | x | | | x | | | | | | | | | | | | | | | | | | | | | | | | | |
| P63162 | | x | | | | | | | | | | | | | | | | | | | | | | | | | | | | |
| Q12799 | | x | | | | | | | | | | | | | | | | | | | | | | | | | | | | |
| Q16637 | | x | | | | | | | | | | | | | | | | | | | | | | | | | | | | |
| Q3KQU3 | | x | | | | | | | | | | | | | | | | | | | | | | | | | | | | |
| Q5T3F8 | | x | | | | | | | | | | | | | | | x | | | | | | | | | | | | | |
| Q5T8P6 | | x | | | | | | | | | | | | | | | | | | | | | | | | | | | | |
| Q5TB30 | | x | | | | | | | | | | | | | | | | | | | | | | | | | | | | |
| Q96D71 | | x | | | | | | | | | | | | | | | | | | | | | | | | | | | | |
| Q14004 | | | x | | x | x | | x | | | x | | | | | | | | | | | | | | | | | x | | |
| Q14839 | | | x | | x | | | | | | | | | | | | | | | | | | | | | | | x | | |
| Q99575 | | | x | | x | | | | | | | | | | | | | | | | | | | | | | | | | |
| Q9C0C2 | | | x | | x | | | | | | | | | | | | | | | | | | | | | | | | | |
| O60271 | | | x | | | | | | | | | | | | | | | | | | | | | | | | | | x | |
| P20585 | | | x | | | | | | | | x | | | | | | | | | | | | | | | | | | | |

| | | | | | | | | | | | | | | | | | | | | | | | | | | | | | | |
|---|---|---|---|---|---|---|---|---|---|---|---|---|---|---|---|---|---|---|---|---|---|---|---|---|---|---|---|---|---|---|
| Q02952 | | | X | | | | | | | | | | | | | | | | | | | | | | | | | | | |
| Q15554 | | | X | | | | | | | | X | | | | | | | | | | | | | | X | | | | | |
| Q9H583 | | | X | | | | | | | | | | | | | | | | | | | | X | | | | | | | |
| Q9NPQ8 | | | X | | | | | | | | | | | | | | | | | | | | | | | | | | | |
| Q9UNF1 | | | X | | | | | | | | | | | | | | | | | | | | | | | | | | | |
| P24557 | | | | X | | | | | | X | | | | | | | | | | | | | | | | | | | | |
| P30837 | | | | X | | | | | | | | | | | | | | | X | | | | | | | | | | | |
| P54619 | | | | X | | | | | | | | | | | | | | | | | | | | | | | | | | |
| Q14914 | | | | X | | | | | | | | | | | | | | | | | | | | | | | | | | |
| Q96Q06 | | | | X | | | | | | | | | | | | | | | | | | | | | | | | | | |
| Q99541 | | | | X | | | | | | | | | | | | | | | | | | | | | | | | | | |
| P35659 | | | | | X | | | X | | | | | | | | | | | | | | | | | | | | | | |
| P42695 | | | | | X | X | | | | | | | | | | | | | | | | | | | X | | | | | |
| Q12974 | | | | | X | X | | | | | | | | | | | | | | | | | | | X | | | | | |
| Q15155 | | | | | X | | | | | | | | | | | | | | | X | | | | | | | | | | |
| Q15287 | | | | | X | | | | | | | | | | | | | | | | | | | | | | | | | |
| Q15477 | | | | | X | | | | | | | | | | | | | | | | | | | | | | | | | |
| Q16527 | | | | | X | | | | | | | | | | | | | | | | | | | | | | | | | |
| Q7KZ85 | | | | | X | | | | | | | | | | | | | | | | | | | | | | | X | | |
| Q7L2E3 | | | | | X | | | | | | | | | | | | | | | | | | | | | | | | | |
| Q7Z5K2 | | | | | X | | | | | | | | | | | | | | | | | | | | | | | | | |
| Q8NBU5 | | | | | X | | | | | | | | | | | | | | | | | | | | X | | | | | |
| Q8TF68 | | | | | X | | | | | | | | | | | | | | | | | | | | | | | X | | |
| Q969S3 | | | | | X | | | | | | | | | | | | | | | | | | | | | | | X | | |
| Q96RK0 | | | | | X | | | | | | | | | | | | | | | | | | | | | | | | | |
| Q9H6F5 | | | | | X | | | | | | | | | | | | | | | | | | | | | | | | | |
| Q9Y5V0 | | | | | X | | | | | | | | | | | | | | | | | | | | | | | | | |
| O00754 | | | | | | | | | | | | | | X | | X | | | | | | | | | | | | | | |
| O14880 | | | | | | | | | | X | | | | | | | | | | | | | | X | | | | | | |
| O15143 | | | | | | | | | X | | | | | | | | | X | | | | | | | | | | | | |
| O15511 | | | | | | | | X | X | | | | | | | | | X | | | | | | | | | | | | |
| O43516 | | | | | | | | | X | | | | | | | | | | | | | | | | | | | | | |
| O75293 | | | | | | | | | | | X | | | | | | | | | X | | | | | X | | | | X | |
| O76039 | | | | | | | | | | | | | | | | | | | | | | | | | X | | | | | |
| O94804 | | | | | | | | | | | | | | | | | | | | | | | | | | | X | | | |
| O95139 | | | | | | | | | | | | | | | | | | | X | | | | | | | | | | | |
| P05362 | | | | | | | | X | | | | | | X | X | | | X | | | | | | | | | | | | |
| P09601 | | | | | | | | | | | | | | | | | | | | | | | | | | | | | X | |
| P10636 | | | | | | | | | X | | | | | | | | | | | | | | | | | | | | | |
| P11279 | | | | | | | | | | | | | | X | | X | X | X | | | | | | | | | | | | |
| P11474 | | | | | | | | | | | | | | | | | | | | | | | | | | | | | X | |
| P17275 | | | | | | | | | | | | | | | | | | | | | | | | | X | | | X | | |
| P17661 | | | | | | | | | X | | | | | | | | | | | | | | | | | | | | | |
| P27105 | | | | | | | | | | | | X | | | | | | | | | | | | | | | | | | |
| P29144 | | | | | | | | | | | | | | | | | | | | | | X | | | | | | | | |
| P30043 | | | | | | | | | | | | | | | | | | | | | | | | X | | | | | | |

| | | | | | | | | | | | | | | | | | | | | | | | | | | | | | | |
|---|---|---|---|---|---|---|---|---|---|---|---|---|---|---|---|---|---|---|---|---|---|---|---|---|---|---|---|---|---|---|
| P35613 | | | | | | | | | | | | | x | x | | | | x | | | x | | | | | | | | | |
| P36957 | | | | | | | | | | | | | | | | | | | x | | | | | | | | | | | |
| P40121 | | | | | | | | x | x | | | | | | | | | | | | | | | | | | | | | |
| P48163 | | | | | | | | | | | | | | | | | | | | | | | | x | | | | | | |
| P49662 | | | | | | | | | | | | | | | | | | | | x | | | | | | | | | | |
| P55786 | | | | | | | | | | | | | | | | | | | | | | | | | x | | | | | |
| P57088 | | | | | | | | | | | | | | | | | x | | | | | | | | | | | | | |
| Q14139 | | | | | | | | | | | | | | | | | | | | | | x | | | | | | | | |
| Q15819 | | | | | | x | | | | | x | | | | | | | | | | | x | | | | | | | | |
| Q16719 | | | | | | | | | | | | | | | | | | | | | | | x | | | | | | | |
| Q6WCQ1 | | | | | | | | | x | | | | | | | | | x | | | | | | | | | | | | |
| Q8IW41 | | | | | | | x | | | | | | | | | | | | | | | | | | | | | | x | x |
| Q8IYD1 | | | | | | | | x | | | | | | | | | | | | | | | x | | | | | | | |
| Q8NEF9 | | | | | | | | | | | | | | | | | | | | | | | | | | | | x | | |
| Q8TBC4 | | | | | | | | | | | | | | | | | | | | | | | | | x | | | | | |
| Q96KB5 | | | | | | | x | | | | | | | | | | | | | | | | | | | | | | | |
| Q9BQ52 | | | | | | | | | | | | | | | | | | | | | | | x | | | | | | | |
| Q9BZ29 | | | | | | | | | | | | | | | | | | x | | | | | | | | | | | | |
| Q9GZS1 | | | | | | | | | | | | | | | | | | | | | | | | | x | | | | | |
| Q9H501 | | | | | | | | | | | | | | | | | | | | | | | | | | | | x | | |
| Q9H853 | | | | | | | | | x | | | | | | | | | | | | | | | | | | | | | |
| Q9NSD9 | | | | | | | | x | | | | | | | | | | | | | | | x | | | | | | | |
| Q9UET6 | | | | | | | | | | | | | | | | | | | | | | | | | | x | | | | |
| Q9UJY1 | | | | | | | x | | | | | | | | | | | | | | | | | | | | | | | |
| Q9Y263 | | | | | | | | | | | | | | | x | | | | | | | | | | | | | | | |
| Q9Y399 | | | | | | | | | | | | | | | | | | | x | | | | | | | x | | | | |

**Table S2 A, B.** Proteins induced by 5µM 15d-PGJ2 in A375 melanoma cells after 48 hours. The proteins are classified by the CPL/MUW database. Uniprot serves as reference for the function of the proteins. In addition, the accession numbers are from the Uniprot database. Numbers indicate distinct peptides identified by mass spectrometry. C: cytoplasm, N: nucleous, S: supernatant.
