# Supplementary material for: Proteome Analysis Identified the PPARγ Ligand 15d-PGJ2 as a Novel Drug Inhibiting Melanoma Progression and Interfering with Tumor-Stroma Interaction
Source: PLoS One. 2012 Sep 25;7(9):e46103. doi: 10.1371/journal.pone.0046103 (PMC3458105; doi:10.1371/journal.pone.0046103)
Supplement: Table S3 — Proteins downregulated by 5 µM 15d-PGJ2 in A375 melanoma cells after 48 hours. Uniprot serves as reference for the function of the proteins. In addition, the accession numbers are from the Uniprot database. Numbers indicate distinct peptides identified by mass spectrometry. (PDF) [file pone.0046103.s004.pdf]

Table S3: Downregulated candidates by 15d-PGJ2

AccNr	Name	Control	15d-PGJ2 (5µM)	Function (Ref. Uniprot)
Q13685	Angio-associated migratory cell protein	1	0	Plays a role in angiogenesis and cell migration.
Q16610	Extracellular matrix protein 1 precursor	1	0	Involved in endochondral bone formation as negative regulator of bone mineralization. Stimulates the proliferation of endothelial cells and promotes angiogenesis.
Q6FI81	Anamorsin	1	0	May be required for the maturation of extramitochondrial Fe/S proteins. Has anti-apoptotic effects in the cell.
P11802	Cell division protein kinase 4	1	0	Probably involved in the control of the cell cycle. Defects in CDK4 are a cause of susceptibility to cutaneous malignant melanoma type 3 (CMM3)
P50613	Cell division protein kinase 7	1	0	Cyclin-dependent kinases (CDKs) are activated by the binding to a cyclin and mediate the progression through the cell cycle.
P78396	Cyclin-A1	1	0	May be involved in the control of the cell cycle at the G1/S (start) and G2/M (mitosis) transitions.
P51946	Cyclin-H	1	0	Regulates CDK7, the catalytic subunit of the CDK-activating kinase (CAK) enzymatic complex. Involved in cell cycle control
P14635	G2/mitotic-specific cyclin-B1	1	0	Essential for the control of the cell cycle at the G2/M (mitosis) transition.
Q9BY12	S phase cyclin A-associated protein in the endoplasmic reticulum	1	0	CCNA2/CDK2 regulatory protein that transiently maintains CCNA2 in the cytoplasm
P43246	DNA mismatch repair protein Msh2	4	1	Component of the post-replicative DNA mismatch repair system (MMR). Forms two different heterodimers: MutS alpha (MSH2-MSH6 heterodimer) and MutS beta (MSH2-MSH3 heterodimer) which binds to DNA mismatches thereby initiating DNA repair
P08253	Matrix metalloproteinase-2	1	0	Ubiquitinous metalloproteinase that is involved in diverse functions such as remodeling of the vasculature, angiogenesis, tissue repair, tumor invasion, inflammation, and atherosclerotic plaque rupture.
P35221	Alpha-1 catenin	2	1	Associates with the cytoplasmic domain of a variety of cadherins. The association of catenins to cadherins produces a complex which is linked to the actin filament network, and which seems to be of primary importance for cadherins cell-adhesion properties. Can associate with both E- and N-cadherins. May play a crucial role in cell differentiation.
P35222	Catenin beta-1	1	0	Involved in the regulation of cell adhesion. The majority of beta-catenin is localized to the cell membrane and is part of E-cadherin/catenin adhesion complexes which are proposed to couple cadherins to the actin cytoskeleton. Ref.32
O94776	Metastasis-associated protein MTA2	1	0	May be involved in the regulation of gene expression as repressor and activator. The repression might be related to covalent modification of histone proteins.
O00560	Syntenin-1	1	0	Seems to function as an adapter protein. In adherens junctions may function to couple syndecans to cytoskeletal proteins or signaling components. Seems to couple transcription factor SOX4 to the IL-5 receptor (IL5RA). May also play a role in vesicular trafficking. Seems to be required for the targeting of TGFA to the cell surface in the early secretory pathway.
Q9NWT1	p21-activated protein kinase-interacting protein 1	3	1	Negatively regulates the PAK1 kinase. PAK1 is a member of the PAK kinase family, which have been shown to play a positive role in the regulation of signaling pathways involving MAPK8 and RELA.
P49023	Paxillin	2	0	Cytoskeletal protein involved in actin-membrane attachment at sites of cell adhesion to the extracellular matrix (focal adhesion).
P08962	Melanoma-associated antigen ME491	1	0	This antigen is associated with early stages of melanoma tumor progression. May play a role in growth regulation.
P43355	Melanoma-associated antigen 1	1	0	Not known, though may play a role in embryonal development and tumor transformation or aspects of tumor progression. Antigen recognized on a melanoma by autologous cytolytic T-lymphocytes.
P62993	Growth factor receptor-bound protein 2	2	0	Adapter protein that provides a critical link between cell surface growth factor receptors and the Ras signaling pathway.
P12004	Proliferating cell nuclear antigen (PCNA)	6	4	This protein is an auxiliary protein of DNA polymerase delta and is involved in the control of eukaryotic DNA replication by increasing the polymerase's processibility during elongation of the leading strand
P46087	Proliferating-cell nucleolar antigen p120	6	3	May play a role in the regulation of the cell cycle and the increased nucleolar activity that is associated with the cell proliferation.
Q9UQ80	Proliferation-associated protein 2G4	13	9	May play a role in a ERBB3-regulated signal transduction pathway. Seems be involved in growth regulation
P06493	Cell division control protein 2 homolog	6	0	Plays a key role in the control of the eukaryotic cell cycle. It is required in higher cells for entry into S-phase and mitosis.

Table S3. Proteins downregulated by 5μM 15d-PGJ2 in A375 melanoma cells after 48 hours. Uniprot serves as reference for the function of the proteins. In addition, the accession numbers are from the Uniprot database. Numbers indicate distinct peptides identified by mass spectrometry.
