# Supplementary material for: Proteome Analysis Identified the PPARγ Ligand 15d-PGJ2 as a Novel Drug Inhibiting Melanoma Progression and Interfering with Tumor-Stroma Interaction
Source: PLoS One. 2012 Sep 25;7(9):e46103. doi: 10.1371/journal.pone.0046103 (PMC3458105; doi:10.1371/journal.pone.0046103)
Supplement: Table S4 — Chaperones regulated by 5 µM 15d-PGJ2 in A375 melanoma cells after 48 hours. The accession numbers are from the Uniprot database. Numbers indicate distinct peptides identified by mass spectrometry. C: cytoplasm, N: nucleous, S: supernatant. (PDF) [file pone.0046103.s005.pdf]

Table S4: Regulated chaperones and Heat shock proteins by 15d-PGJ2

AccNr	Name	control	15-PGJ2	Δ	SM-Scor	Fraction
P11142	Heat shock cognate 71 kDa protein	34	19	-15	565.32	C, S, N
P08238	Heat shock protein HSP 90-beta (HSP 90) (HSP 84)	33	18	-15	528.87	C, S, N
P07900	Heat shock protein HSP 90-alpha (HSP 86)	33	20	-13	544.13	C, S, N
P11021	78 kDa glucose-regulated protein (GRP 78)	23	13	-10	365.75	C, S, N
P17987	T-complex protein 1 subunit alpha (TCP-1-alpha)	20	10	-10	320.94	C, N
P49368	T-complex protein 1 subunit gamma (TCP-1-gamma) (CCT-gamma) (hTRiC5)	15	5	-10	230.97	C, N
P10809	60 kDa heat shock protein, mitochondrial (Heat shock protein 60) (HSP-60)	36	28	-8	648.87	C, N
P14625	Endoplasmin (Heat shock protein 90 kDa beta member 1)	17	9	-8	238.31	C, N
P30101	Protein disulfide-isomerase A3 (Disulfide isomerase ER-60)	11	3	-8	154.12	C, N
P48643	T-complex protein 1 subunit epsilon	18	10	-8	268.49	C, N
P50990	T-complex protein 1 subunit theta (TCP-1-theta)	17	9	-8	249.51	C, N
Q92598	Heat shock protein 105 kDa (Heat shock 110 kDa protein)	15	8	-7	219.83	C, N
P78371	T-complex protein 1 subunit beta (TCP-1-beta)	23	16	-7	365.17	C, N
P38646	Stress-70 protein, mitochondrial (75 kDa glucose-regulated protein)	13	7	-6	201.73	C, N
P34932	Heat shock 70 kDa protein 4	15	10	-5	240.88	C, N
P04792	Heat shock protein beta-1 (HspB1) (Heat shock 27 kDa protein) (HSP 27)	8	3	-5	108.88	C, N
P50991	T-complex protein 1 subunit delta (TCP-1-delta) (CCT-delta) (Stimulator of TAR RNA-binding)	15	10	-5	264.13	C, N
Q99832	T-complex protein 1 subunit eta (TCP-1-eta)	16	11	-5	253.33	C, N
P61604	10 kDa heat shock protein, mitochondrial (Hsp10)	7	3	-4	109.65	C, S, N
P40227	T-complex protein 1 subunit zeta (TCP-1-zeta)	19	15	-4	346.23	C, N
P27797	Calreticulin (CRP55) (Calregulin)	5	2	-3	81.21	C, N
P08107	Heat shock 70 kDa protein 1 (HSP70.1) (HSP70-1/HSP70-2)	15	12	-3	228.12	C, N
P07237	Protein disulfide-isomerase (PDI)	9	6	-3	149.75	C, N
Q58FF8	Putative heat shock protein HSP 90-beta 2	6	3	-3	96.45	C, N
Q9NQP4	Prefoldin subunit 4 (Protein C-1)	4	2	-2	67.02	C, N
Q99471	Prefoldin subunit 5 (C-myc-binding protein Mm-1)	5	3	-2	96.61	C, N
P30040	Endoplasmic reticulum protein ERp29	8	7	-1	109.52	C, N
P34931	Heat shock 70 kDa protein 1L	6	5	-1	107.92	C, N
P61758	Prefoldin subunit 3	3	2	-1	50.65	C, N
P13667	Protein disulfide-isomerase A4	3	2	-1	40.84	C, N
Q15084	Protein disulfide-isomerase A6	8	7	-1	124.13	C, N
Q96JJ7	Protein disulfide-isomerase TMX3		1	-1	11.91	C
Q96MM6	Heat shock 70 kDa protein 12B	1	1	0	12.1	N
P17066	Heat shock 70 kDa protein 6	4	4	0	70.85	C, N
O60925	Prefoldin subunit 1	1	1	0	16.45	C, N
Q9UHV9	Prefoldin subunit 2	4	4	0	60.91	C, S, N
O15212	Prefoldin subunit 6	5	5	0	64.16	C, N

Table S4: Chaperones regulated by 5µM 15d-PGJ2 in A375 melanoma cells after 48 hours. The accession numbers are from the Uniprot database. Numbers indicate distinct peptides identified by mass spectrometry. C: cytoplasm, N: nucleous, S: supernatant.
